# Dietary Leucine - An Environmental Modifier of Insulin Resistance Acting on Multiple Levels of Metabolism

**DOI:** 10.1371/journal.pone.0021187

**Published:** 2011-06-22

**Authors:** Yazmin Macotela, Brice Emanuelli, Anneli M. Bång, Daniel O. Espinoza, Jeremie Boucher, Kirk Beebe, Walter Gall, C. Ronald Kahn

**Affiliations:** 1 Joslin Diabetes Center and Harvard Medical School, Boston, Massachusetts, United States of America; 2 Metabolon, Inc., Durham, North Carolina, United States of America; University of Las Palmas de Gran Canaria, Spain

## Abstract

Environmental factors, such as the macronutrient composition of the diet, can have a profound impact on risk of diabetes and metabolic syndrome. In the present study we demonstrate how a single, simple dietary factor—leucine—can modify insulin resistance by acting on multiple tissues and at multiple levels of metabolism. Mice were placed on a normal or high fat diet (HFD). Dietary leucine was doubled by addition to the drinking water. mRNA, protein and complete metabolomic profiles were assessed in the major insulin sensitive tissues and serum, and correlated with changes in glucose homeostasis and insulin signaling. After 8 weeks on HFD, mice developed obesity, fatty liver, inflammatory changes in adipose tissue and insulin resistance at the level of IRS-1 phosphorylation, as well as alterations in metabolomic profile of amino acid metabolites, TCA cycle intermediates, glucose and cholesterol metabolites, and fatty acids in liver, muscle, fat and serum. Doubling dietary leucine reversed many of the metabolite abnormalities and caused a marked improvement in glucose tolerance and insulin signaling without altering food intake or weight gain. Increased dietary leucine was also associated with a decrease in hepatic steatosis and a decrease in inflammation in adipose tissue. These changes occurred despite an increase in insulin-stimulated phosphorylation of p70S6 kinase indicating enhanced activation of mTOR, a phenomenon normally associated with insulin resistance. These data indicate that modest changes in a single environmental/nutrient factor can modify multiple metabolic and signaling pathways and modify HFD induced metabolic syndrome by acting at a systemic level on multiple tissues. These data also suggest that increasing dietary leucine may provide an adjunct in the management of obesity-related insulin resistance.

## Introduction

Obesity and type 2 diabetes are determined by the interplay between genetics and environmental influences. Diet, physical activity, intrauterine environment and even social factors have been shown to impact on genetic background and alter metabolic homeostasis [1], [2]. With regard to diet, most attention has focused on how changes in macronutrient composition, i.e., the proportion of fat, carbohydrate and protein, can affect metabolic disease risk. However, how individual nutrients may act as metabolic regulators is less clear.

The branched chained amino acids (BCAA) leucine, isoleucine and valine have been shown to function as regulators of hormonal signaling in addition to serving as nutrients. High protein diets (HPD), a source of BCAA, have been shown to be beneficial for weight loss and reduce glucose concentrations in type 2 diabetes patients [3]–[7]. In some studies, however, HPDs increase fasting glucose, primarily through impairment of insulin suppression of hepatic glucose output [8]. At the molecular level, BCAA, especially leucine, can activate the mammalian Target Of Rapamycin (mTOR) leading to activation of p70S6 kinase and increased serine phosphorylation of IRS-1 [9], which inhibits insulin signaling and insulin-stimulated glucose transport in muscle [10] and fat [11]. Recently, Newgard et al showed that administration of a mixture of BCAA to rats on a HFD increased insulin resistance [12]. On the other hand, leucine has been shown to rescue insulin signaling in adipose tissue explants obtained from insulin resistant db/db mice [13].

In the present study we have examined how a single dietary BCAA, leucine, alters metabolism and insulin signaling in a mouse model of insulin resistance and metabolic syndrome, namely HFD-induced obesity. We show that despite its effects to activate p70S6K, a two-fold increase in dietary leucine improves glucose tolerance, prevents hepatic steatosis, reduces obesity-induced adipose tissue inflammation and rescues insulin signaling in muscle, liver and fat. Furthermore, increasing dietary leucine restores many abnormalities in the metabolomic profile of serum and tissues of mice on HFD, illustrating how a single environmental factor can influence multiple tissues and multiple metabolic pathways to alter the development of type 2 diabetes and the metabolic syndrome.

## Results

### Leucine supplementation of mice on chow (CD) and high fat diets (HFD)

**Figure 1:**
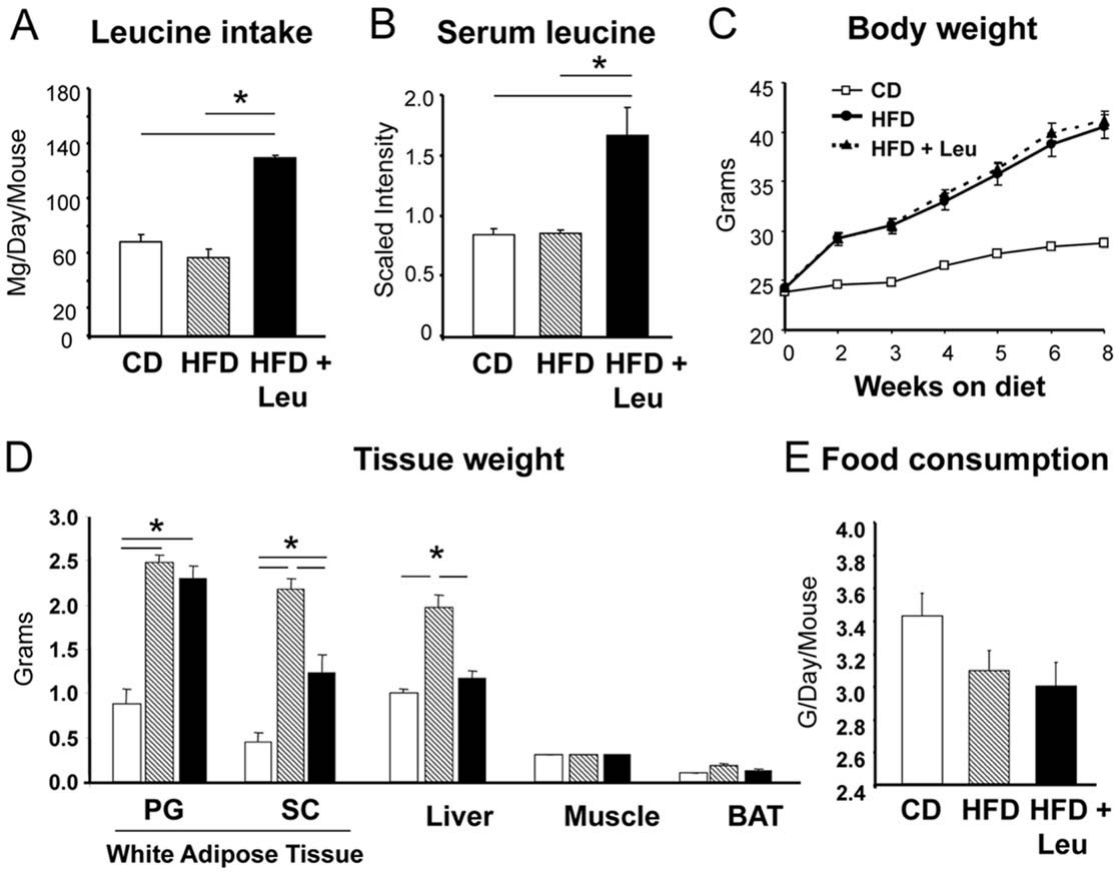
Effect of dietary leucine on body weight, fat mass and food consumption. Eight week-old male C67BL/6 mice were fed a chow diet or a high fat diet, with or without supplementation by leucine (1.5% w/v) in the drinking water for 8 weeks as described in Methods. A) Leucine intake was calculated based on the amount of leucine consumed in the drinking water and the amount contained in the food as provided by the manufacturers (25 mice per group). B) Histogram plots showing relative levels of serum leucine as measured by UHPLC-MS/MS at the end of the eighth week with 8–10 mice per group. C) Body weights were monitored at the weeks indicated (25 mice per group). D) Tissue weight was measured at the end of the 8 weeks on each diet (5 mice per group). E) Food intake was measured every week and the mean consumption was calculated for every dietary intervention (25 mice per group).

Mice were placed on either a regular CD or HFD, without or with supplemental leucine (1.5% w/v) in the drinking water. This resulted in a doubling of leucine intake from 60–70 mg/day to 130 mg/day (Figure 1A) and an approximate doubling of serum leucine levels (Figure 1B), without affecting food or caloric intake (Figure 1E).

As expected, HFD mice increased their body weights 40% more than CD mice over the 8 weeks of study and had increases in perigonadal (PG) and subcutaneous (SC) fat pad and liver weight, reflecting increased obesity and hepatosteatosis (Figure 1D). Mice on HFD+Leu had similar increases in body weight (Figure 1C) and PG fat pad weight (Figure 1D) and showed no significant difference in total fat or lean mass on DXA scanning (Figure S1). However, upon sacrifice, mice on HFD+Leu had a significantly smaller increase in SC fat pad weight than mice on HFD alone (2.7- vs. 4.7-fold, p<0.05) and did not show the 2-fold increase in liver weight observed in HFD mice (Figure 1D) (P<0.05). These changes occurred with no difference in food consumption between mice on HFD and HFD+Leu (Figure 1E). Metabolic cage assessment showed no differences in energy expenditure or heat production in HFD+Leu mice compared to HFD (Figure S2). Likewise, serum adiponectin, leptin, triglycerides, glucagon and c-peptide levels were similar between HFD and HFD+Leu fed mice (Figure S3). Addition of leucine to mice on CD had no effect on weight gain, organ weight or any of the physiological parameters studied (examples shown in Figure S4). Therefore, in subsequent sections, data on the effects of leucine in CD mice is presented only with regard to effects on metabolite profiles.

### Leucine supplementation improved glucose tolerance and reduced hepatic steatosis

**Figure 2:**
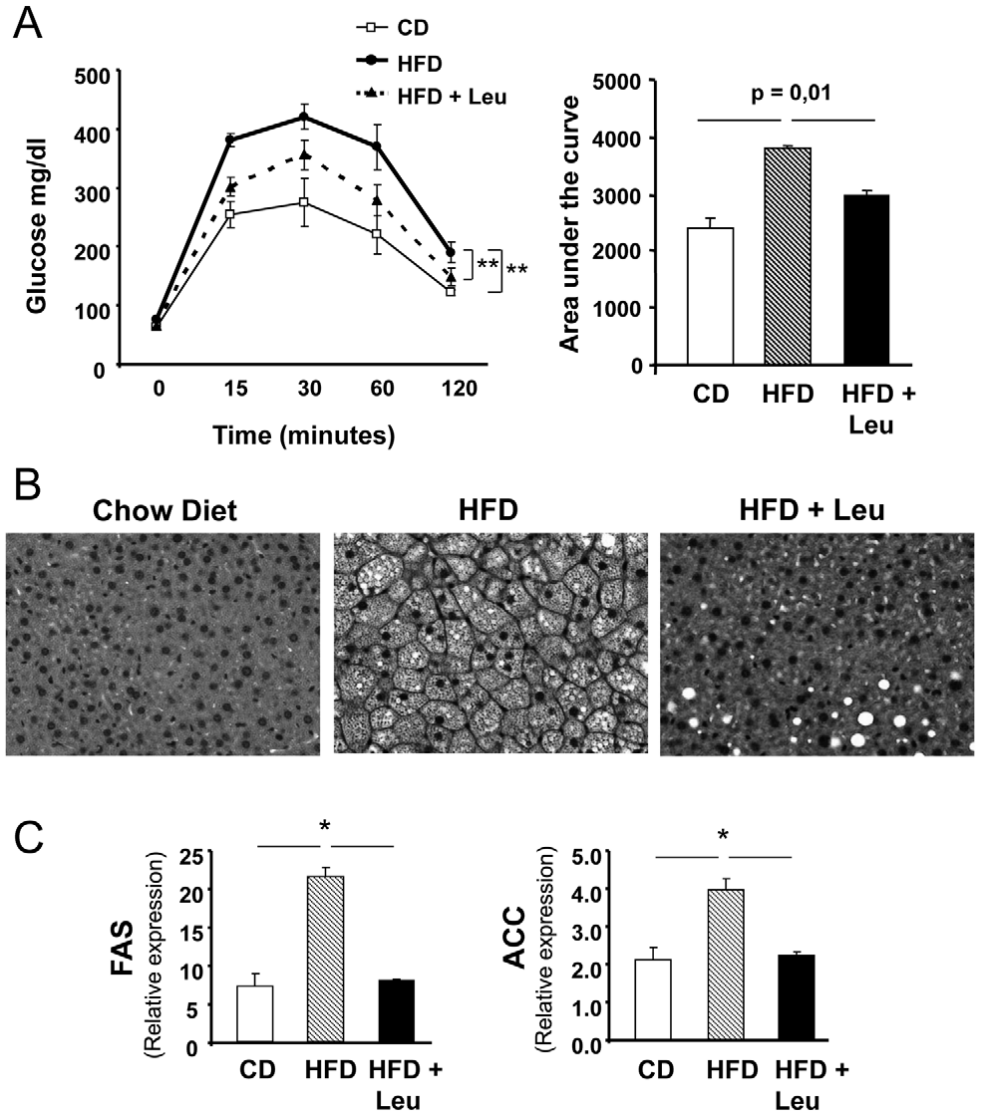
Leucine supplementation improves glucose tolerance and hepatic steatosis in mice on HFD. A) Glucose tolerance tests were performed after an overnight fast in the three study groups. Mice received an intraperitoneal injection of 2 g/kg body wt glucose. Glucose was measured in tail vein blood samples at the indicated times. The area under the curve (AUC) was calculated for each dietary condition. Data points are means ± SEM with 7–8 mice in each group. B) Mice were sacrificed and livers were harvested after 8 weeks on each diet. The livers were then formalin fixed, embedded in paraffin and sections stained with H&E. Three mouse livers per group were analyzed. C) mRNA was extracted from 200 mg liver and subjected to quantitative real time PCR. Gene expression for FAS and ACC were normalized against TATA-binding protein (TBP). 5 livers per group were used. *P<0.05.

As expected, mice on a HFD showed markedly impaired glucose tolerance (GT) compared to CD mice with peak glucoses of 420±21 mg/dl versus 274±41 mg/dl, respectively (Figure 2A). Addition of Leu to the HFD, on the other hand, showed significantly improved GT compared to mice on HFD alone with peak glucoses of 356±25 mg/dl (p<0.05 vs HFD alone) (Figure 2A). The improved glucose tolerance could also be observed by calculation of the area under the curve (AUC) during the GTT, with a 50% increase in AUC of the HFD mice compared to the CD mice and an intermediate 25% increase in area in HFD+Leu mice compared to mice on CD (p = 0.01 between HFD and HFD+Leu) (Figure 2A). Random fed glucose levels were also lower in the HFD+Leu mice compared to HFD, but there was no difference in the glucose lowering effect of insulin during the insulin tolerance test (Figure S5). Histological examination of liver revealed marked intracellular lipid accumulation in mice on HFD compared to controls, and this was markedly reduced in mice on HFD+Leu (Figure 2B), consistent with decreased expression of lipogenic enzymes observed under these conditions. For example,expression of lipogenic enzymes fatty acid synthase and acetyl CoA carboxylase mRNA levels were increased 3-fold and 2-fold, respectively, in livers of HFD mice compared to CD mice, and these reverted to control levels in the HFD+Leu mice (Figure 2C). Leucine supplementation also helped normalize expression of glucokinase and pyruvate kinase, and reduced expression of inflammation markers genes: TNFα, F4/80 and CD68 in liver (Figure S6).

### Effect of leucine supplementation on insulin signaling, p70S6K and AMP Kinase

**Figure 3:**
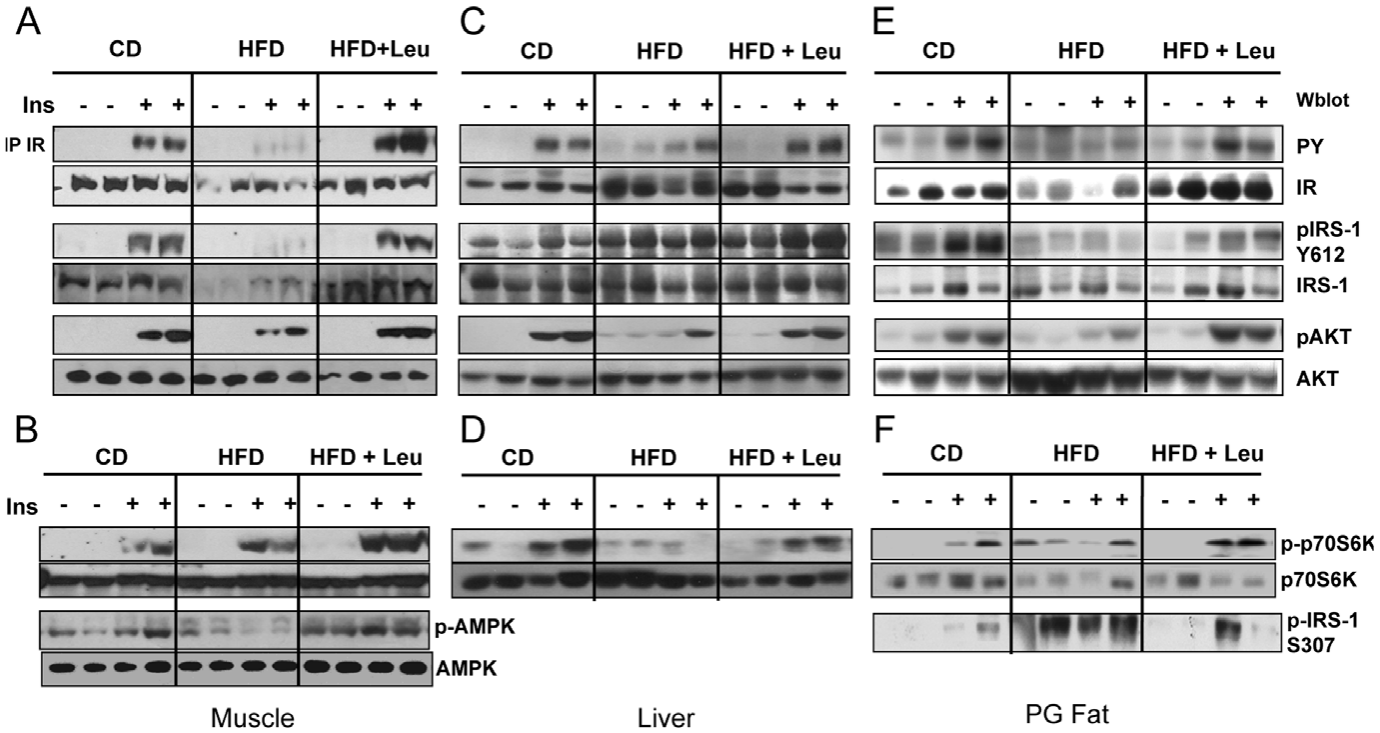
Leucine supplementation rescues insulin signaling in muscle, liver and fat and stimulates p70S6K and AMPK phosphorylation. After 8 weeks on the different diets, mice were injected i.v. with insulin (5 U per mouse) or saline, and tissues were harvested 5 minutes later. Tissue protein lysates (20 µg) were run on SDS-PAGE and subjected to western blot using antibodies directed against phosphorylated or total p70S6K, IRS-1 and AKT. Insulin receptor was immunoprecipitated with anti-IR antibody and blotted for phosphotyrosine 4G10 or insulin receptor (ß-subunit). Panels A and B show the muscle data; panels C and D, the liver data; and panels E and F, the fat data. IRS-1 phosphorylated on Ser 307 was also assessed in fat lysates (panel F), and AMPK phosphorylated on T172 was assesed in muscle lysates (panel B) by western blotting. 5 animals per group were used, the experiments and the blots were repeated 2 times.

To determine if the effect of leucine to improve glucose tolerance was due to improved insulin sensitivity, we analyzed the insulin signaling pathway in muscle, liver and adipose tissue. In the control CD mice, i.v. insulin injection resulted in a robust increase in phosphorylation of the insulin receptor (IR), IRS-1 and AKT in muscle, liver and fat (Figures 3A, C, E and Figure S7). In HFD mice, these insulin effects were markedly blunted. In muscle and fat, there was almost no detectable increase in IR and IRS-1 tyrosine phosphorylation and a 50% decrease in AKT serine phosphorylation (Figures 3A and 3E). Likewise, in liver of HFD mice, there was >50% decrease in tyrosine phosphorylation of IRS-1 and IR and a 40% decrease in insulin stimulated serine phosphorylation of AKT (Figure 3C). Addition of leucine to the HFD almost completely rescued the insulin-stimulation of IR, IRS-1 and AKT, returning them to levels similar to those in the CD mice (Figures 3A, 3C and 3E). HFD-induced insulin resistance also caused a decrease in IR and IRS-1 protein in muscle by 60% and 64%, respectively (Figure 3A) and a reduction of IR protein in fat by 48% (Figure 3E and Figure S8), whereas AKT protein levels were increased upon HFD feeding (Figure 3E). All these changes also normalized in the HFD mice supplemented with leucine (Figures 3A, 3C, 3E and Figure S8, also extra loading controls for all tissues in Figure S9).

Leucine acts together with insulin to increase protein synthesis in muscle via the activation of the mTOR/p70S6K pathway [14]. To determine if leucine supplementation could increase the activation of p70S6K, Western blots were performed on muscle, liver and fat extracts. In the basal state, p70S6K phosphorylation was not detectable in any of the mice. Following insulin stimulation, there was a robust increase in the phosphorylation of p70S6K in muscle and liver of CD mice (Figures 3B and 3D). In liver, insulin-stimluated phosphorylation of p70S6K was reduced in HFD mice and returned toward normal in HFD+Leu mice (Figure 3D). In muscle, insulin-stimulated p70S6k was enhanced in HFD mice and further enhanced in the HFD+Leu mice, indicating the effects of both HFD and leucine to potentiate insulin-stimulated p70S6K activation (Figure 3B).

Increases in IRS-1 Ser307 phosphorylation have been shown to reflect activation of stress kinases in many insulin resistant states [15]. Ser307 phosphorylation of IRS-1 was barely detectable in fat from CD mice and increased 5.4-fold in mice on HFD (Figure 3F and Figure S8). This phosphorylation was observed in both the basal and insulin stimulated state. Surprisingly, despite the increased phosphorylation/activation of p70S6K, fat from mice fed a HFD+Leu had reduced IRS-1 Ser307 phosphorylation.

AMPK can act as an insulin sensitizer in muscle and therefore improve the general metabolic profile. As expected, AMPK phosphorylation was decreased in muscle of HFD mice compared to CD fed animals, and leucine rescued its phosphorylation (Figure 3B). This occurred independent of insulin stimulation.

### Effect of dietary leucine on adipose tissue morphology and inflammation

**Figure 4:**
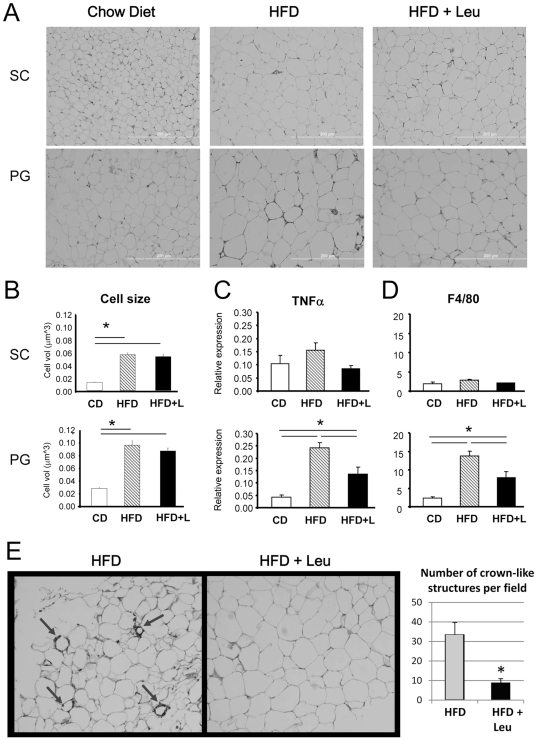
Effects of HFD and leucine on adipose tissue morphology, macrophage content and inflammatory markers. After 8 weeks on each diet, mice were sacrificed and perigonadal (PG) and subcutaneous (SC) adipose tissues were harvested, formalin fixed and paraffin embedded. A and B) Sections were H&E stained and cell size calculated measuring the area of 100 individual cells in three fields per slide, in three different tissues per group for each adipose depot. C and D) mRNA was extracted from PG and SC fat and subjected to quantitative real time PCR for inflammatory markers TNFα and F4/80. Five samples per group were used. *P<0.05. E) Perigonadal adipose tissues from HFD and HFD+Leu mice were also paraffin embedded, and sections processed for immunohistochemistry using F4/80 antibody to stain for macrophages. 3 samples per group were analyzed and showed similar results. Quantifications were calculated from 3 different fields from 3 different mice per group. *P<0.05.

Despite the 35% reduction in weight of the SC fat pad, adipocyte size in both SC and PG fat did not change in HFD+Leu compared to HFD mice (Figures 4A and 4B). As expected, there were “crown-like” structures in the PG fat from HFD mice characteristic of increased macrophage infiltration and tissue inflammation [16]. HFD also increased the expression of the pro-inflammatory cytokine TNFα and the macrophage marker F4/80 in the PG fat by ∼6 -fold compared to mice fed a CD (Figures 4C and 4D). Addition of leucine to the HFD blocked appearance of the macrophage infiltrates (Figure 4A) and reduced the levels of TNFα and F4/80 by 40–45%. This was confirmed by immunohistochemistry of the PG fat using F4/80 antibody with a decrease in the number of crown-like structures from 33.3 per field in HFD to 7.7 per field in HFD+Leucine mice (Figure 4E). These changes were also reflected in changes in expression of genes important for adipocyte function. Thus, adiponectin and GLUT4 were decreased in HFD and normalized to control levels in HFD+Leu mice (Figure S10). Expression of FAS and ACC did not change.

### Effects of dietary leucine on metabolomic profiles

To define the changes in metabolites that might contribute to leucine's positive effects on insulin sensitivity and metabolism, we performed an unbiased, comprehensive metabolomic profiling of serum, liver, perigonadal fat and muscle of mice on CD, CD+Leu, HFD and HFD+Leu. This revealed changes not only in leucine and its metabolites, but in a broad range of metabolites in multiple metabolic pathways of protein, lipid and carbohydrate metabolism.

#### Leucine metabolism

**Figure 5:**
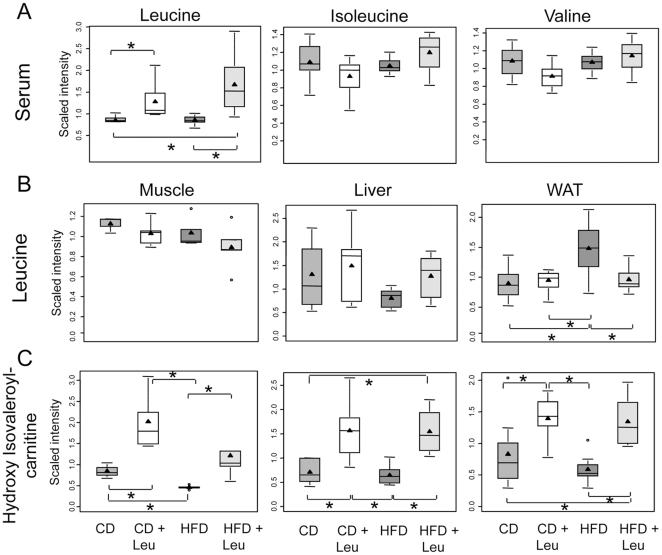
Changes in leucine metabolites in serum, muscle, liver and fat. After 8 weeks on each diet, serum, hindlimb skeletal muscle, liver and perigonadal fat were obtained, extracted and subjected to non-targeted metabolomic analysis by UHPLC-MS/MS and GC-MS (Metabolon). Box-and-whisker plots of the relative levels of (A) serum leucine, isoleucine, and valine, (B) Leucine levels in muscle, liver and perigonadal fat, (C) Leucine catabolite hydroxyisovaleroylcarnitine metabolite levels in muscle, liver and perigonadal fat. 3–9 samples per group were used. *P<0.05. The X-axis shows the four groups (CD, CD+Leu, HFD, HFD+Leu) and the Y-axis shows the relative normalized intensity for the metabolites measured. Within the boxplot, the mean value is represented by the black arrowhead, the median by the horizontal dividing line, and the top and bottom of the box represent the seventy-fifth and twenty-fifth percentile, with the whiskers indicating the maximum and minimum points and outlier points shown as small circles.

Ingestion of HFD vs. CD had no effect on serum BCAA levels, but doubling the dietary intake of leucine increased serum leucine levels in both the CD+Leu and HFD+Leu groups by 1.5- to 2-fold (p≤0.05) without changes in serum levels of isoleucine and valine (Figure 5A). Interestingly, the levels of leucine and its metabolites in tissues were quite different from those observed in serum. Thus, leucine levels in skeletal muscle were not changed by leucine supplementation, but levels of the leucine metabolite hydroxyisovaleroyl-carnitine (HIV-carnitine) were increased 2.3-fold (p≤0.05) in CD+Leu, reduced to 46% of CD (p≤0.05) by HFD and restored to slightly above control levels in HFD+Leu mice (Figures 5B and 5C). Likewise in liver, leucine levels were not changed in CD + Leu mice, while the level of HIV-carnitine was increased 2.2-fold (p≤0.05). Interestingly, levels of both leucine metabolites in liver were significantly reduced by HFD (61% and 90%, respectively, p≤0.05), and addition of leucine to the HFD restored lecuine levels to normal and increased HIV-carnitine levels by 2.2-fold (p≤0.05). By contrast, leucine levels in PG fat increased in mice on HFD and were reduced toward normal in HFD+Leu mice. The leucine metabolite HIV-carnitine was increased by 2.3-fold (p≤0.05) in PG fat from CD+Leu mice, tended to decrease on HFD mice (0.7-fold) and increased on HFD+Leu mice (1.6-fold, p = 0.05) compared to levels in CD mice (Figures 5B and 5C).

#### Glucose and energy metabolism

**Figure 6:**
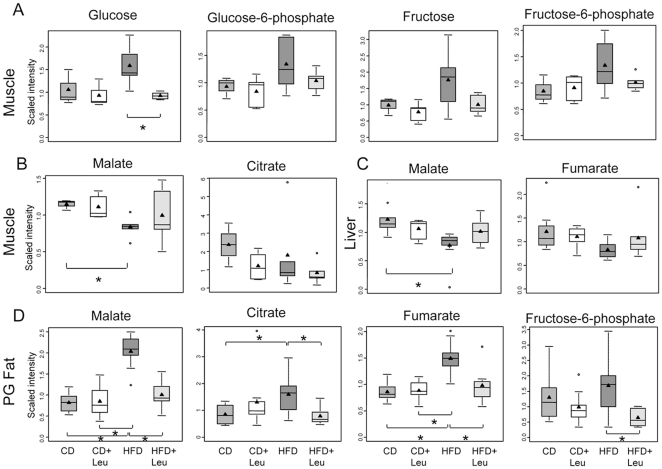
Changes in glucose and TCA cycle metabolites in muscle, liver and fat. After 8 weeks on each diet, serum, hindlimb skeletal muscle, liver and perigonadal fat were obtained, extracted and subjected to non-targeted metabolomic analysis by UHPLC-MS/MS and GC-MS (Metabolon). Box-and-whisker boxplots of relative levels are shown for A) Glucose and glucose metabolites in muscle, B) TCA cycle metabolites in muscle, C) TCA cycle metabolites in liver, and D) TCA cycle metabolites and fructose-6-phosphate in perigonadal. 3–9 samples per group were used. *P<0.05.

In addition to increasing serum glucose, HFD increased glucose and glucose metabolites (glucose 6-phosphate, fructose and fructose 6-phosphate) in muscle as compared to CD by 1.4- to 1.6-fold, and levels of all these metabolites returned to normal in HFD+Leu mice (Figure 6A). Tissue levels of several TCA cycle intermediates were also perturbed by HFD and normalized by leucine treatment. For example, fumarate was reduced in livers of HFD mice and tended to normalize in HFD + Leu (Figures 6B and 6C). Likewise, malate levels tended to be reduced 30–40% in both muscle and liver from HFD fed mice compared to CD mice and were normalized by leucine supplementation (Figures 6B and 6C), while the level of citrate in muscle was reduced in HFD and was further reduced in HFD + Leu. In perigonadal fat, on the other hand,TCA cycle intermediates (malate, citrate, fumarate), as well as fructose-6-phosphate, were significantly increased by 1.3–2.5 fold in HFD vs. CD mice. Addition of leucine to the HFD restored all of these metabolites to the levels found in CD mice (Figure 6D).

#### Fatty Acids

HFD resulted in increases in serum levels of several long chain free fatty acids LC-FFA), including arachidonate, dihomolinoleate, eicosatrienoate and adrenate, by 1.3- to 2.5-fold (all p<0.05). Surprisingly, the serum levels of the latter three were increased further by 30–60% in mice on HFD+Leu, despite the general improvement in metabolism. Levels of the majority of the remaining LC-FFA were decreased in HFD mice, and most were restored toward normal in mice on HFD+Leu (Table 1). The most dramatic changes in tissue FFA were observed in the perigonadal fat, where half of the FFA species analyzed were increased in the HFD group. Again, most of these were restored toward normal in the HFD+Leu group (Table 1).

**Table 1:** Fatty Acid Levels in Serum and Perigonadal Fat.

SERUM					
Fatty Acids	Fatty Acid	HFD/Chow	HFD+ Leu/Chow	HFD/Chow	HFD+Leu/Chow
				P-VALUE	P-VALUE
**Essential fatty acids**	Docosapentaenoate (n3 DPA; 22:5n3)	0.55	0.82	0.0001	0.1917
	Docosahexaenoate (DHA; 22:6n3)	0.65	0.84	0.0000	0.0867
**Long chain fatty acid**	Palmitate (16:0)	0.73	0.88	0.0001	0.1507
	Margarate (17:0)	0.82	1.06	0.0224	0.5128
	10-heptadecenoate (17:1n7)	0.67	0.85	0.0102	0.3118
	Oleate (18:1n9)	0.84	1.03	0.0297	0.7254
	Linoleate (18:2n6)	0.75	0.88	0.0004	0.1284
	Nonadecanoate (19:0)	0.68	0.95	0.0189	0.6420
	10-nonadecenoate (19:1n9)	0.57	0.78	0.0009	0.2374
	Eicosenoate (20:1n9 or 11)	0.64	0.93	0.0053	0.6917
	Dihomo-linoleate (20:2n6)	1.34	1.79	0.0236	0.0001
	Eicosatrienoate (20:3n9)	1.96	3.05	0.0180	0.0000
	Arachidonate (20:4n6)	2.48	3.23	0.0000	0.0000
	Adrenate (22:4n6)	1.48	2.23	0.0024	0.0000

PERIGONADAL FAT					
	Fatty Acid	HFD/Chow	HFD+ Leu/Chow	HFD/Chow	HFD+Leu/Chow
				P-VALUE	P-VALUE
**Essential fatty acid**	Linolenate [alpha or gamma; (18:3n3 or 6)]	1.41	0.99	0.0324	0.9274
**Medium chain fatty acid**	Laurate (12:0)	2.02	0.91	0.0297	0.2381
	5-dodecenoate (12:1n7)	5.04	1.16	<0.001	0.5021
**Long chain fatty acid**	Myristate (14:0)	1.75	0.96	0.0100	0.9505
	Myristoleate (14:1n5)	3.86	1.20	<0.001	0.5122
	Pentadecanoate (15:0)	2.28	0.75	0.0550	0.6313
	Palmitate (16:0)	1.26	0.97	0.0618	0.9933
	Palmitoleate (16:1n7)	1.59	0.92	0.0026	0.6022
	Heptadecanoate (17:0)	3.65	1.02	0.0043	0.7948
	Linoleate (18:2n6)	1.20	0.94	0.0862	0.7844

Free Fatty Acids were analyzed by metabolomics in serum and fat from mice after 8 weeks on each diet. Essential, medium and long chain fatty acids are represented. Differences between HFD and CD as well as HFD + Leu and CD (ratios) are shown iserum (top panel) and perigonadal fat (bottom panel).

#### Cholesterol and bile acid and sterol metabolism

**Figure 7:**
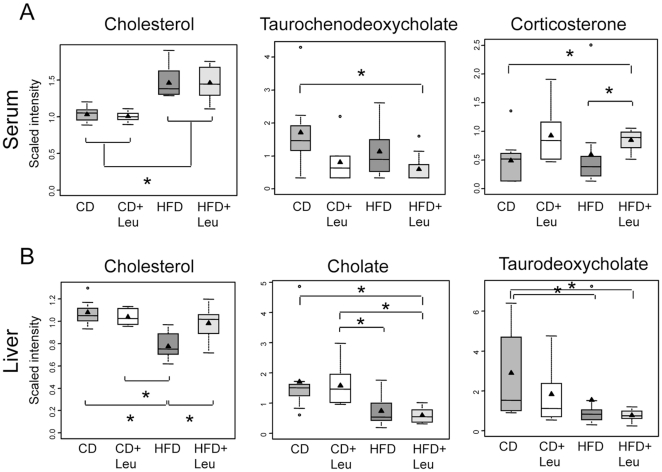
Changes in cholesterol, bile acids and sterol metabolism in serum and liver. After 8 weeks on each diet, serum and liver were obtained, extracted and subjected to non-targeted metabolomic analysis by UHPLC-MS/MS and GC-MS (Metabolon). Box-and-whisker plots showing relative levels of A) serum sterol, bile acid, and steroid levels, and B) liver cholesterol and bile acid metabolites in sterol-bile acid biosynthetic pathway. 3–9 samples per group were used. *P<0.05.

Serum cholesterol levels were increased in mice on HFD 1.4-fold (p≤0.05) and not changed by leucine supplementation in either CD or HFD mice. Cholesterol can be metabolized into bile acids and steroid hormones. Interestingly, the level of the serum bile acid taurochenodeoxycholate was reduced by leucine supplementation in both CD+Leu and HFD+Leu mice compared to their controls (Figure 7A). This reduction mirrored an increase in serum corticosterone levels induced by leucine treatment (CD+Leu, 1.4-fold; HFD+Leu, 1.7-fold) (Figure 7A). In contrast to the changes in serum, cholesterol levels in the liver were reduced to 72% of control in mice on HFD (p≤0.05), and these levels increased toward normal in HFD+Leu mice (Figure 7B).

#### Other pathways related to liver energy metabolism

Nicotinamide adenine dinucleotide (NAD+), a cofactor for energy production and the action of the sirtuin protein deacetylases, was reduced in liver (and muscle) from HFD mice to 74% of control levels (p≤0.05), and was restored to normal levels by leucine supplementation. Tryptophan, a precursor of NAD+, was also reduced in livers from HFD mice (p<0.05) and restored to normal levels in HFD+Leu mice (Figure S11). Fructose and sorbitol tended to increase in livers of mice on HFD and return towards normal in HFD+Leu. The changes suggest an increase in polyol pathway activity induced by HFD, a pathway involved in development of diabetic complications [17]. Most of these changes were reduced by addition of leucine to the diet (Figure S11).

Finally, levels of α-hydroxybutyrate (α-HB) were found to be elevated in liver from HFD mice and restored to normal by leucine. In serum, α-hydroxybutyrate levels also increased upon HFD but were not normalized by leucine supplementation, despite the fact that leucine reduced the serum levels of α-HB in mice on a CD (Figure S11). In humans, we have shown that among metabolites in the circulation, α-hydroxybutyrate shows the best correlation with insulin resistance and serves as an early marker of glucose intolerance [18].

## Discussion

In the present study we sought to determine how a single, minimal environmental change, i.e., a modest increase in the intake of one amino acid, could impact on metabolic homeostasis and insulin resistance. We chose the essential BCAA leucine, since is not synthesized in the body and is obtained entirely through dietary intake. Leucine is abundant in all protein food sources [19] and is also interesting physiologically, since it regulates mTOR signaling [20], [21] and impacts on several metabolic processes [22].

We show that increasing dietary leucine by as little as two-fold can have an impact on insulin signaling, tissue macrophage infiltration and the entire metabolic profile of an animal. While the changes in metabolite profile induced by leucine are similar in both CD and HFD mice, the physiological effects are most profound in the context of HFD, where doubling dietary leucine reduces HFD-induced insulin resistance, inflammatory changes in adipose tissue, glucose intolerance and hepatic steatosis, without modifying weight gain. Leucine's effect to reduce the hepatic steatosis induced by HFD occurs via reduced expression of lipogenic genes, and, like the effects on glucose metabolism, is independent of changes in adiposity. Similarly, in humans, high protein diets have shown to decrease hepatic lipid deposition without altering body weight or adiposity [23].

The data herein demonstrate that improvement of glucose tolerance and hepatic steatosis by leucine supplementation correlates with improved insulin signaling in muscle, liver and fat. This includes enhancement in the phosphorylation/activation of the insulin receptor, IRS-1 and AKT. This is in contrast to the *in vitro* effects of leucine to increase mTOR-p70S6K mediated serine phosphorylation of IRS-1 resulting in decreased insulin signaling and insulin action in muscle, fat and liver [24]. We find that despite the increase in activation of p70S6K in HFD+Leu mice compared to HFD mice, which *in vitro* results in increased serine phosphorylation and decreased tyrosine phosphorylation of IRS-1 [25]–[27], the increased serine phosphorylation of IRS-1 *in vivo* produced by leucine supplementation is associated with improved insulin signaling and increased tyrosine phosphorylation of IRS-1. Whether this reflects a difference between *in vitro* and *in vivo* activities or differences between insulin resistant and non-insulin resistant conditions is unclear, but the observations reported here are in agreement with Hinault et al [13], [28] who found that BCAA treatment can rescue AKT activation in insulin resistant adipocytes from ob/ob mice or adipocyte cell lines, in which PI 3-kinase activity has been blocked by wortmannin.

Consistent with effects of hyperosmotic stress to also contribute to the development of insulin resistance and diabetes [29], [30], we observe sorbitol and downstream intermediates (fructose, fructose-6-phosphate) accumulating in the liver in HFD mice (Figure S11). When glycolytic capacity is reduced such as in muscle and liver (Figure 6 and Figure S11) under insulin resistant conditions, glucose is redistributed to other glucose utilization pathways such as sorbitol. Sorbitol serves as a energy storage repository in overnutrition conditions, and such excess nutrient availability has been associated with increases in the S6K1 kinase pathway. The hyperosmotic stress that occurs when sorbitol is elevated in tissues has been shown to contribue to the reduction in insulin sensitivity [31].

We also found that AMPK phosphorylation, which is reduced in muscle of mice on HFD, is rescued to control levels by leucine supplementation. This change could help explain a number of components in the improvement in insulin sensitivity and metabolism in these mice.

This may also reflect a difference between chronic and acute effects of amino acid exposure. For example, short-term treatment of rats with BCAA produces insulin resistance [12], while long-term BCAA supplementation has been shown to improve insulin resistance in patients with liver disease [32]. Human studies have also shown that high protein diet, which provides increases in dietary leucine, can reduce glycemia in patients with type 2 diabetes without effect on body weight [33], but this is, at least in part, due to improved insulin secretion [6].

Some of the differences between in vivo and in vitro studies may reflect the fact that in vivo effects of leucine on one tissue can impact other tissues and hence on whole body metabolic homeostasis. For example, leucine had no effects on size of the PG fat pad or PG adipocytes, but leucine supplementation was able to block the macrophage infiltration and expression of inflammation markers in PG fat from mice fed a HFD, which in turn would improve insulin resistance in other tissues and enhance whole body glucose metabolism. Exactly how leucine modifies the inflammation in adipose tissue without modifying adipocyte hypertrophy remains to be determined. Amino acids are essential for the increase in protein synthesis needed for adequate immune system function [34]. Rapamycin, an mTOR inhibitor, has been extensively used as an immunosupressant and suppressor of T-cell proliferation [35]. However, rapamycin can also have inflammatory side effects [36]. Leucine is an activator of mTOR, and the mTOR pathway has been shown to reduce inflammation in monocytes [37]. Thus, dietary leucine may inhibit adipose inflammation via mTOR inhibition of NFkB, a transcription factor that has been shown to be an important regulator of adipose tissue inflammation and participant in the development of insulin resistance [38].

Exactly how leucine and other nutrients exert effects on metabolism is complex and may depend on the dietary context, species and dose of the nutrient. Consistent with our studies, Zhang, et al [39] and Ropelle, et al [40] have reported improved metabolism and glucose tolerance in obese mice and rats following leucine supplementation. Some of these effects of leucine occurred at higher levels of supplementation, which resulted in reduced food intake and reduced obesity. On the other hand, Nairizi et al [41] observed improved glucose levels in HFD fed mice given leucine supplementation without changes in food consumption, body weight, or adiposity. Although there was no significant change in body weight in our HFD+Leu group compared to HFD alone, there was a significant reduction in subcutaneous fat mass, a feature not analyzed in other studies. Previous studies from our lab and others have shown that subcutaneous fat may have beneficial effects on insulin sensitivity and metabolism [42].

Another comparison to our study is the recent study by Newgard, et al [12], which showed that rats fed a HFD together with supplemental BCAA (150% increase of valine, leucine and isoleucine) became even more insulin resistant than rats fed HFD only, despite the fact that adding BCAA to the diet resulted in reduced food intake and reduced weight gain. In addition to the differences between species, it is interesting to note that feeding the mixture of BCAA resulted in increases in serum levels of all three BCAA, whereas feeding leucine only, increased the level of leucine in serum, but did not change in serum levels of valine and isoleucine. This difference suggests that increasing the levels of the other BCAA may contribute to insulin resistance observed.

Metabolomic analysis showed changes in multiple metabolic pathways driven by leucine supplementation. Thus, while serum leucine did not change in HFD, some of its metabolites did, and leucine supplementation normalized levels of these metabolites. In tissues, HFD results in an increase in leucine levels in fat, but no change in muscle and a reduction in liver. However, the leucine metabolite hydroxyl isovaleroyl-carnitine was reduced (or tended to be reduced) in all tissues from HFD animals, suggesting decreased leucine metabolism, and these levels were increased to above normal by leucine supplementation in both CD and HFD. This C5 acylcarnitine is an intermediate in fatty acid oxidation and could reflect impaired fatty acid oxidation in HFD that is restored by leucine treatment. C3 and C5 acylcarnitines are metabolites of BCAA catabolism, so an increase in dietary leucine can normalize these levels in HFD fed mice. Recently Muoio, et al [43] proposed that insulin resistance in skeletal muscle is linked to an excess, rather than a reduction, in β-oxidation. They showed decreases in muscle and serum C3 and C5 acylcarnitines, but increases in long chain C8 to C16 species in rats on HFD. Newgard et al, also found that in rats fed a HFD, the serum C3, C5 acylcarnitines were reduced compared to controls on a standard diet and that these levels were increased in rats fed a HFD supplemented with BCAA, reaching levels similar to those of control rats. These changes in acylcarnitines C3 and C5 are in agreement with the findings in our study, but in their case, the BCAA-treated animals were insulin resistant, whereas our leucine supplemented animals are rescued from many of the deleterious effects of HFD. This suggests that acylcarnitine levels alone are not a good marker of insulin resistance, since animals on HFD have reduced C3 and C5 levels and are insulin resistant, whereas animals supplemented with leucine or BCAA have levels similar to animals on chow diet and can be insulin sensitive (if leucine supplemented) or insulin resistant (if BCAA supplemented). C3 and C5 acylcarnitines are metabolites of BCAA catabolism, so an increase in dietary leucine can normalize these levels in serum and muscle from HFD fed mice. In favor of a positive role of high levels of acylcarnitine, Lechmann et al. showed that medium chain acylcarnitines are increased during moderately intense exercise and support muscle fat oxidation [44], showed that medium chain acylcarnitines identified by metabolomics are increased during moderately intense exercise and they support muscle fat oxidation, the authors propose that increased acylcarnitines might be part of the beneficial mechanism of excercise to increase beta-oxidation.

Despite impaired glucose tolerance, glucose metabolism, as estimated by intracellular glucose metabolites, is increased in muscle in HFD obese mice and normalized by leucine supplementation. Amelioration of the accumulation of such glycolytic intermediates in the HFD+Leu condition parallels the observed improvement in glucose tolerance and demonstrates that glycolysis efficiency has been restored to control levels. Moreover, in HFD animals, TCA cycle metabolites are decreased in both muscle and liver, indicating impaired metabolism, and these are restored to normal by leucine supplementation. Also, fatty acids accumulated in fat in animals fed a HFD, and these changes were normalized by leucine supplementation. Thus, leucine supplementation restores normal glucose and energy metabolism in tissues of HFD fed mice.

Of particular interest is the cholesterol and bile acid pathway. Serum cholesterol levels were not changed by leucine, but cholesterol levels in liver were reduced in HFD fed animals and normalized with leucine supplementation. Serum corticosterone was not changed in HFD mice, but was increased with leucine supplementation in both CD and HFD. This increased corticosterone may contribute to the effects of leucine to reduce the inflammatory response in adipose tissue which occurred upon HFD feeding.

In conclusion, increasing the dietary content of a single amino acid, leucine, by as little as two-fold can ameliorate many of the deleterious effects of HFD, including adipose tissue inflammation, hepatic lipid deposition and insulin resistance. Leucine supplementation also improves insulin signaling and multiple aspects of the metabolic profile, as well as decreases inflammation in adipose tissue and decreases ectopic lipid deposition in liver. These data demonstrate the complex nature of environmental effects in creating risk for diabetes and metabolic syndrome and point to the importance of a systems biology approach to understanding, not just the effects of macronutrients, but the effects of individual nutrient components on disease pathogenesis.

## Materials and Methods

### Ethics Statement

Protocols for animal use were approved by the Animal Care Use Committee of the Joslin Diabetes Center in accordance with NIH guidelines. Protocol numbers: 1989-23 and 2007-02.

### Animals, dietary conditions and physiological measurements

Eight week old C57BL/6J mice (Jackson Labs) were divided in four groups of 25 and fed a chow diet (5020, Lab Diets) containing 21% of calories from fat or a HFD containing 60% calories from fat (D12492 Research Diets) with or without supplemental leucine (1.5% w/v) (Sigma) in the drinking water. GTT was performed 3 and 8 weeks after starting the diets. Insulin signaling was carried out by injecting mice with 5 U of insulin or saline in the vena cava and harvesting tissues five minutes later.

### Western blot and Immunoprecipitation

200 mg of tissue were used to prepare protein extracts of muscle, liver and perigonadal fat. 20 µg of protein extracts from each tissue were subjected to SDS-PAGE, and blots probed with antibodies to AKT, phospho-AKT [Ser473], phospho-p70S6K [Thr389], phospho AMPK [Thr172], AMPK (Cell Signaling), IRS-1 (BD Transduction), phospho-IRS-1 [Y612] (Biosource), insulin receptor, p70S6K (Santa Cruz) and phosphotyrosine [4G10] (Upstate) (all antibodies diluted 1∶1000). Detection was with horseradish peroxidase-coupled secondary antibodies (at a 1∶5,000 dilution) and enhanced chemiluminescence. Immunoprecipitation of the IR was carried out using 0.5 mg of protein lysates, 1 µg of anti-insulin receptor antibody and protein A/G agarose.

### mRNA analysis and histology

Total RNA was extracted from tissues (Rneasy, QIAGEN). Reverse transcription was performed using 0.5–1 µg of RNA, and quantitative real-time PCR was performed using an ABI 7900 with SYBR Green. Tissues were embedded in paraffin, sectioned, and stained with hematoxylin and eosin. Immunohistochemistry for macrophages was performed with F4/80 antibody (Abcam) and a Vector Peroxidase Kit.

### Sample preparation and metabolomics data acquisition

Briefly, small molecule metabolites were extracted, and the reconstituted extracts were resolved using mass spectrometry platforms, comprising UHPLC-LC-MS/MS and GC-MS. Chromatographic separation of all ions in each sample was followed by library matching of these ions to Metabolon's reference library of standards (>2000 authentic standards, plus thousands of additional library entries of unknown biochemicals based on unique characteristics of retention time, nominal mass and fragmentation pattern). The identity of metabolites was determined by matching the combination of chromatographic retention index and mass spectra signatures compared to the reference library entries. The total number of biochemicals detected and measured for each biological matrix were: serum, 398 metabolites; liver, 444 metabolites; muscle, 257 metabolites; and perigonadal fat, 169 metabolites. Relative quantitation was based on peak integration and expressed in figures as scaled intensity.

### Metabolomic Profiling Platform

The untargeted metabolic profiling platform employed for this analysis was based on a combination of three independent platforms: ultrahigh performance liquid chromatography/tandem mass spectrometry (UHPLC/MS/MS) optimized for basic species, UHPLC/MS/MS optimized for acidic species, and gas chromatography/mass spectrometry (GC/MS), with details of this platform described extensively in a previous publication [45].

Various standards spiked into each sample allowed for estimations of overall process variation and ensure data quality [45]. The median relative standard deviation (RSD) for the internal standards for serum, liver, muscle, and perigonadal fat was 6%, 7%, 9%, and 8%, respectively, reflecting a very low degree of instrument variability. Overall process variability was determined by calculating the median RSD for all endogenous metabolites (i.e., non-instrument standards) present in 100% of a technical replicate sample that consisted of pooled client samples for serum, liver, muscle, and perigonadal fat. Indicative of acceptable process variability, the median RSD for these samples was 13% for liver and muscle and 15% for serum and perigonadal fat.

### Metabolomic Data Normalization and Imputation

The counts for the integrated peak areas for each metabolite in each sample were normalized to correct for variation resulting from instrument inter-day tuning differences. For each metabolite, the raw area counts were divided by its median value for each run-day, therefore setting the medians equal to 1 for each day's run. In this way, the variation between instrument run day is removed, while the variation that exist across experimental samples (e.g. from the course of day 0 to day 15) remains. Missing values (i.e. from an absence of a peak in a particular sample) were imputed with the observed minimum after the normalization step. Data were log transformed for statistical analysis.

### Statistical Analysis

All data are reported as mean ± S.E.M. Comparisons were made using Student's *t* test when comparing two groups and ANOVA for more than two groups. ANOVA repeated meassures test was used for the GTT and ITT analysis. P<0.05 was considered significant.

## Supporting Information

Figure S1 **Lean mass and total body mass are not altered by leucine supplementation.** Lean mass and fat mass were evaluated by Dual energy X-ray absorptiometry (DEXA) in 5 mice per group after 8 weeks on each diet. *P<0.05.
(PPT)

Figure S2 **Leucine supplementation does not change energy expenditure or activity measured by CLAMS.** Metabolic cage studies were performed over a 24 hour period following 1 day of acclimation in 8 mice per group. Mice were fed from 0–24 hrs and fasted from 24–47.5 hrs.
(PPT)

Figure S3 **Leucine supplementation does not change leptin, adiponectin, triglyceride, glucagon or c-peptide serum levels.** Serum levels were evaluated by ELISA in 5 samples per group in random fed animals at 8 weeks after the initiation of each dietary condition. Values are means ± SE.
(PPT)

Figure S4 **Leucine supplementation in chow diet does not change metabolic parameters.** Body weight, GTT, liver histology and liver gene expression were analyzed in mice on a CD, CD+Leu, HFD and HFD+Leu.
(PPT)

Figure S5 **Reduced random fed glucose levels but no change in insulin tolerance test upon leucine supplementation.** Insulin tolerance test was evaluated in random fed animals by i.p. injection of 1 U/kg BW insulin in 7 animals per group. *P<0.05.
(PPT)

Figure S6 **Leucine supplementation normalizes some alterations in liver gene expression induced by HFD.** mRNA was extracted from 200 mg liver and subjected to quantitative real time PCR. Gene expression was normalized against TATA-binding protein (TBP). 5 livers per group were used. *P<0.05 vs CD.
(PPT)

Figure S7 **Leucine supplementation normalizes phosphorylation of IR, IRS1Y and AKT in muscle, liver and PG fat.** Phosphorylation was evaluated by western blot using specific antibodies for each protein. Quantification was done with Quantity one software (BioRad). Graphs represent the fold change in phosphorylation stimulated by insulin vs the non treated control and normalized by densities of the total proteins. N = 5 samples per group were quantified and western blots were repeated two times.*P<0.05 vs CD.
(PPT)

Figure S8 **Leucine supplementation normalizes protein expression of IR and reduces IRS1 S307 phosphorylation in fat and also normalizes IRS1 in muscle.** Protein expression of IRS1 in muscle, IR in PG fat and IRS1 S307 phosphorylation was evaluated by western blot and quantified using Kodak software. n = 5 samples per group.*P<0.05.
(PPT)

Figure S9 **Loading controls for Western blot on liver, muscle and PG fat.** After 8 weeks on the different diets, mice were injected i.v. with insulin (5 U per mouse) or saline, and tissues were harvested 5 minutes later. Tissue protein lysates (20 mg) were run on SDS-PAGE and subjected to western blot using antibodies directed against b-actin for liver and muscle or b-tubulin for PG fat. 5 animals per group were used and the experiments were repeated 2 times and the blots were repeated 2 times.
(PPT)

Figure S10 **Leucine supplementation normalizes some alterations in metabolic visceral fat gene expression induced by HFD.** mRNA was extracted from 200 mg perigonadal fat and subjected to quantitative real time PCR. Gene expression was normalized against TATA-binding protein (TBP). 5 fat depots per group were used. *P<0.05 vs CD.
(PPT)

Figure S11 **Other changes in metabolites induced by leucine supplementation.** After 8 weeks on each diet, serum, hindlimb skeletal muscle, liver and perigonadal fat were obtained, extracted and subjected to non-targeted metabolomic analysis by UHPLC-MS/MS and GC-MS (Metabolon). Box-and-whisker boxplots of relative levels are shown for A) NADH and Tryptophan metabolism, B) Polyol Pathway, C) Alpha-hydroxybutyrate in liver and serum. 3-9 samples per group were used. *P<0.05.
(PPT)
