# Supplementary figures and images for: Dietary Leucine - An Environmental Modifier of Insulin Resistance Acting on Multiple Levels of Metabolism
Source: PLoS One. 2011 Jun 22;6(6):e21187. doi: 10.1371/journal.pone.0021187 (PMC3120846; doi:10.1371/journal.pone.0021187)

## Slide 1
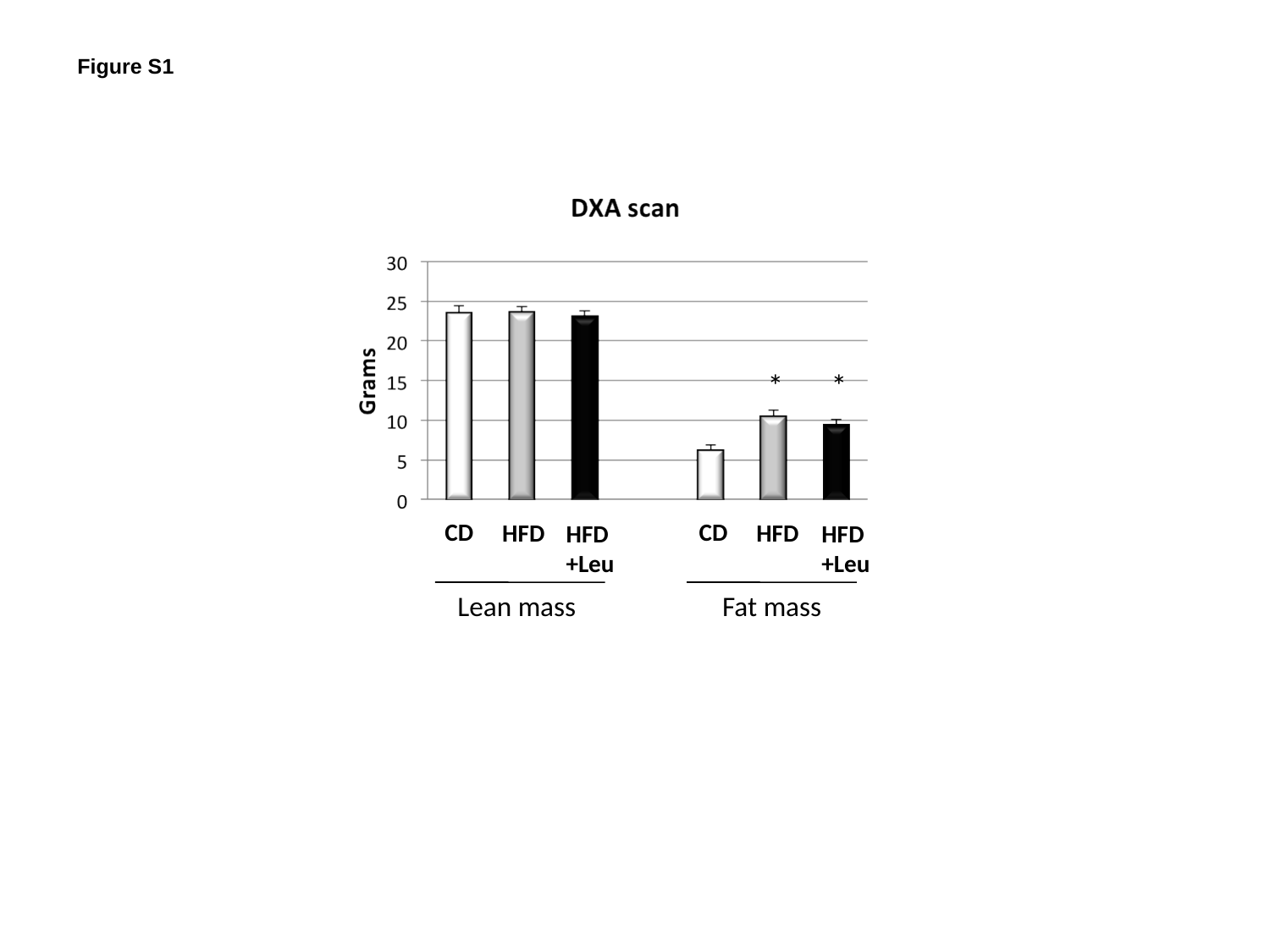

Figure S1
*
*
CD
CD
HFD
HFD
HFD
+Leu
HFD
+Leu
Lean mass
Fat mass

Supplement: Figure S1 — Lean mass and total body mass are not altered by leucine supplementation. Lean mass and fat mass were evaluated by Dual energy X-ray absorptiometry (DEXA) in 5 mice per group after 8 weeks on each diet. *P<0.05. (PPT) [file pone.0021187.s001.ppt]

## Slide 1
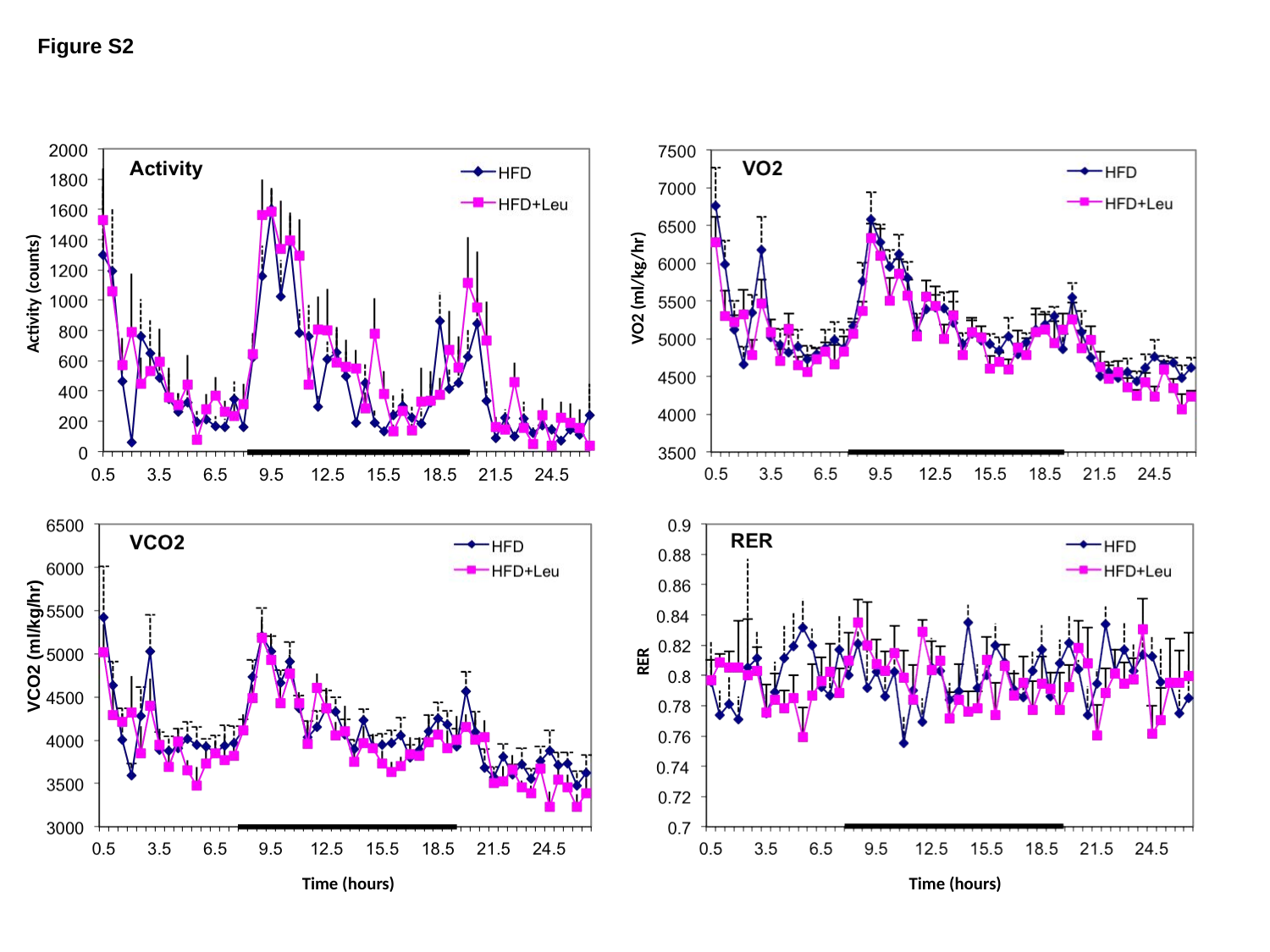

Figure S2
Activity (counts)
VO2 (ml/kg/hr)
VCO2 (ml/kg/hr)
RER
Time (hours)
Time (hours)

Supplement: Figure S2 — Leucine supplementation does not change energy expenditure or activity measured by CLAMS. Metabolic cage studies were performed over a 24 hour period following 1 day of acclimation in 8 mice per group. Mice were fed from 0–24 hrs and fasted from 24–47.5 hrs. (PPT) [file pone.0021187.s002.ppt]

## Slide 1
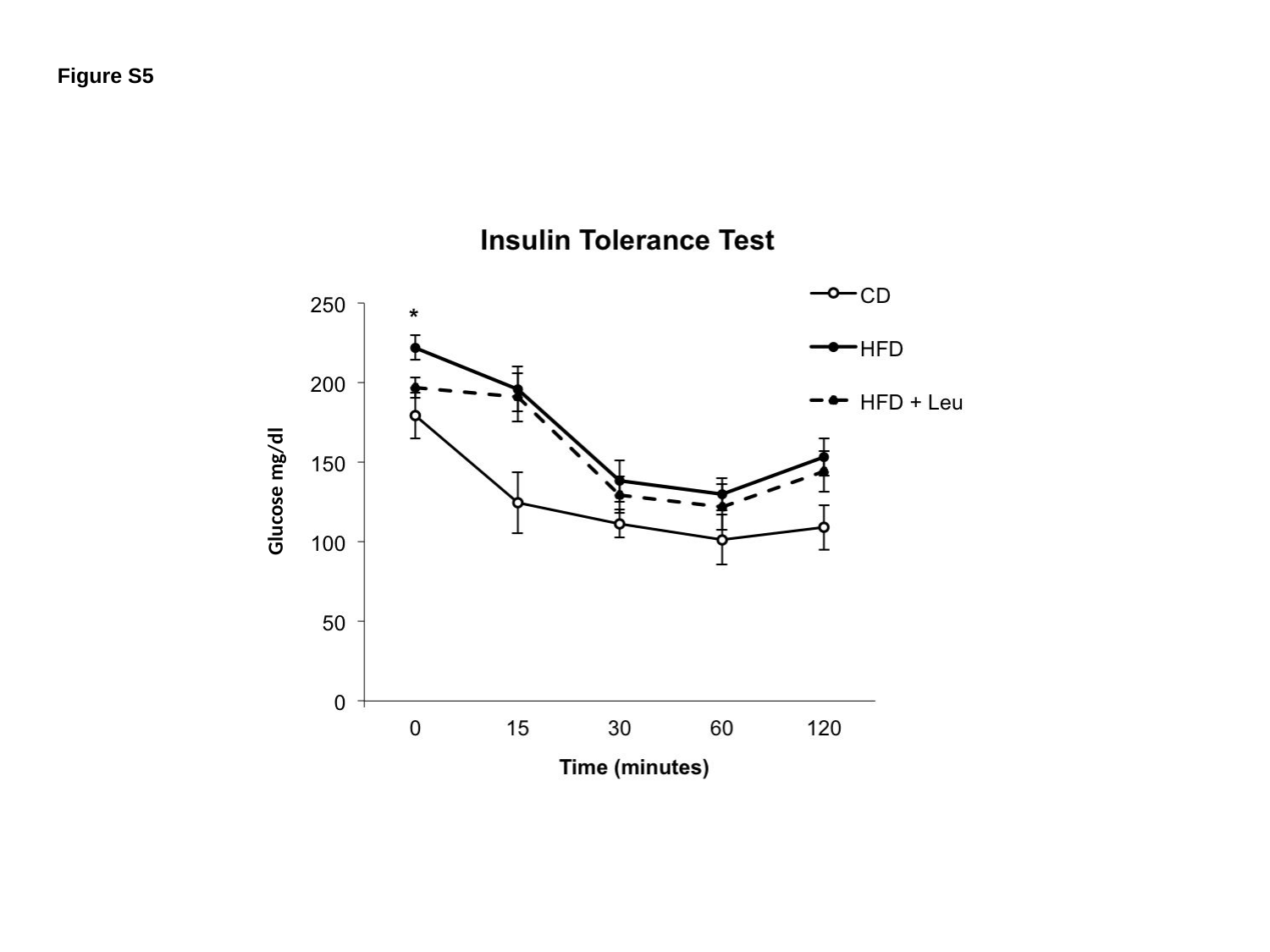

Figure S5
Glucose mg/dl

Supplement: Figure S5 — Reduced random fed glucose levels but no change in insulin tolerance test upon leucine supplementation. Insulin tolerance test was evaluated in random fed animals by i.p. injection of 1 U/kg BW insulin in 7 animals per group. *P<0.05. (PPT) [file pone.0021187.s005.ppt]

## Slide 1
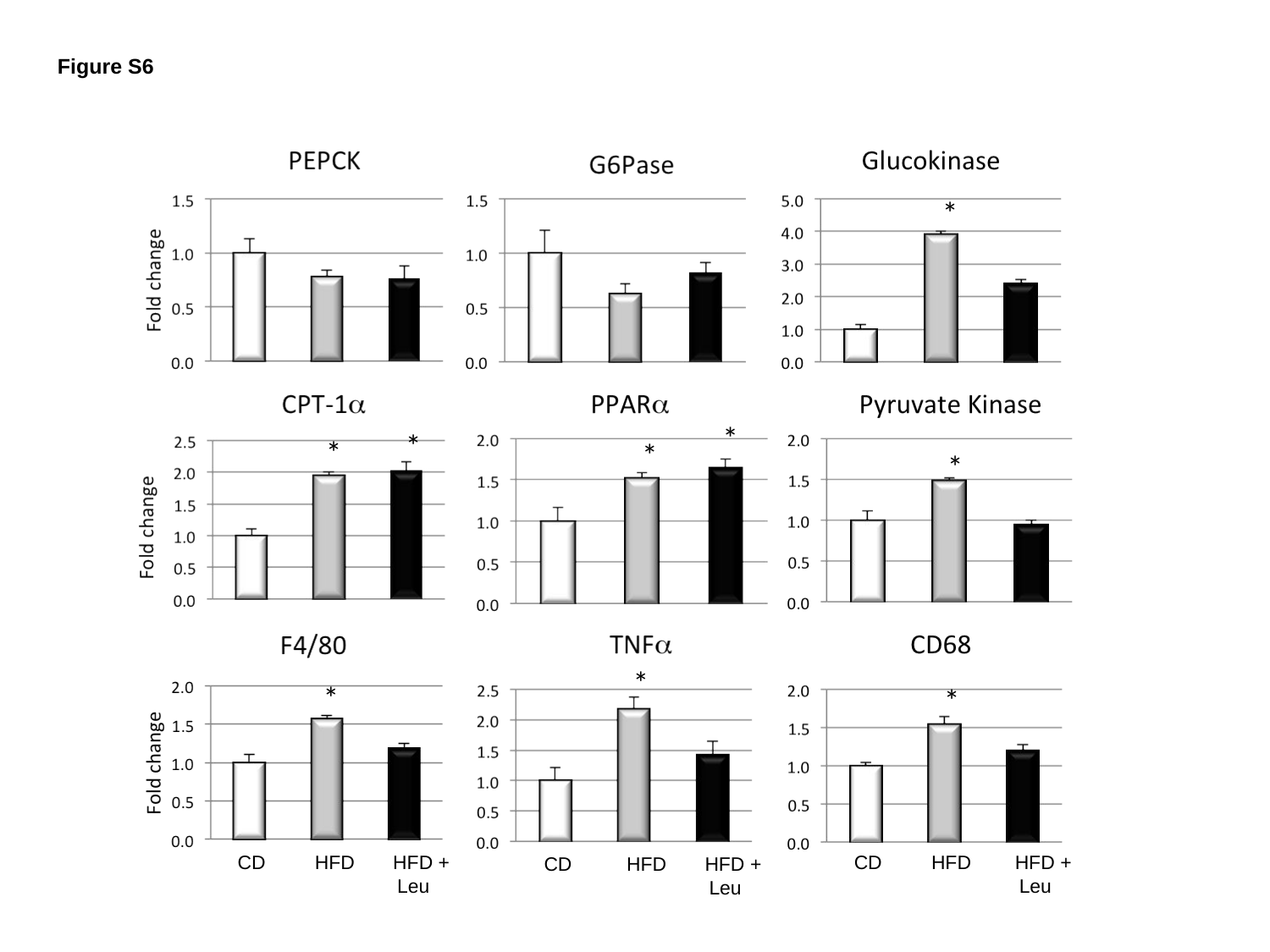

Figure S6
*
*
*
*
*
*
*
*
*
CD HFD HFD +
 Leu
CD HFD HFD +
 Leu
CD HFD HFD +
 Leu

Supplement: Figure S6 — Leucine supplementation normalizes some alterations in liver gene expression induced by HFD. mRNA was extracted from 200 mg liver and subjected to quantitative real time PCR. Gene expression was normalized against TATA-binding protein (TBP). 5 livers per group were used. *P<0.05 vs CD. (PPT) [file pone.0021187.s006.ppt]

## Slide 1
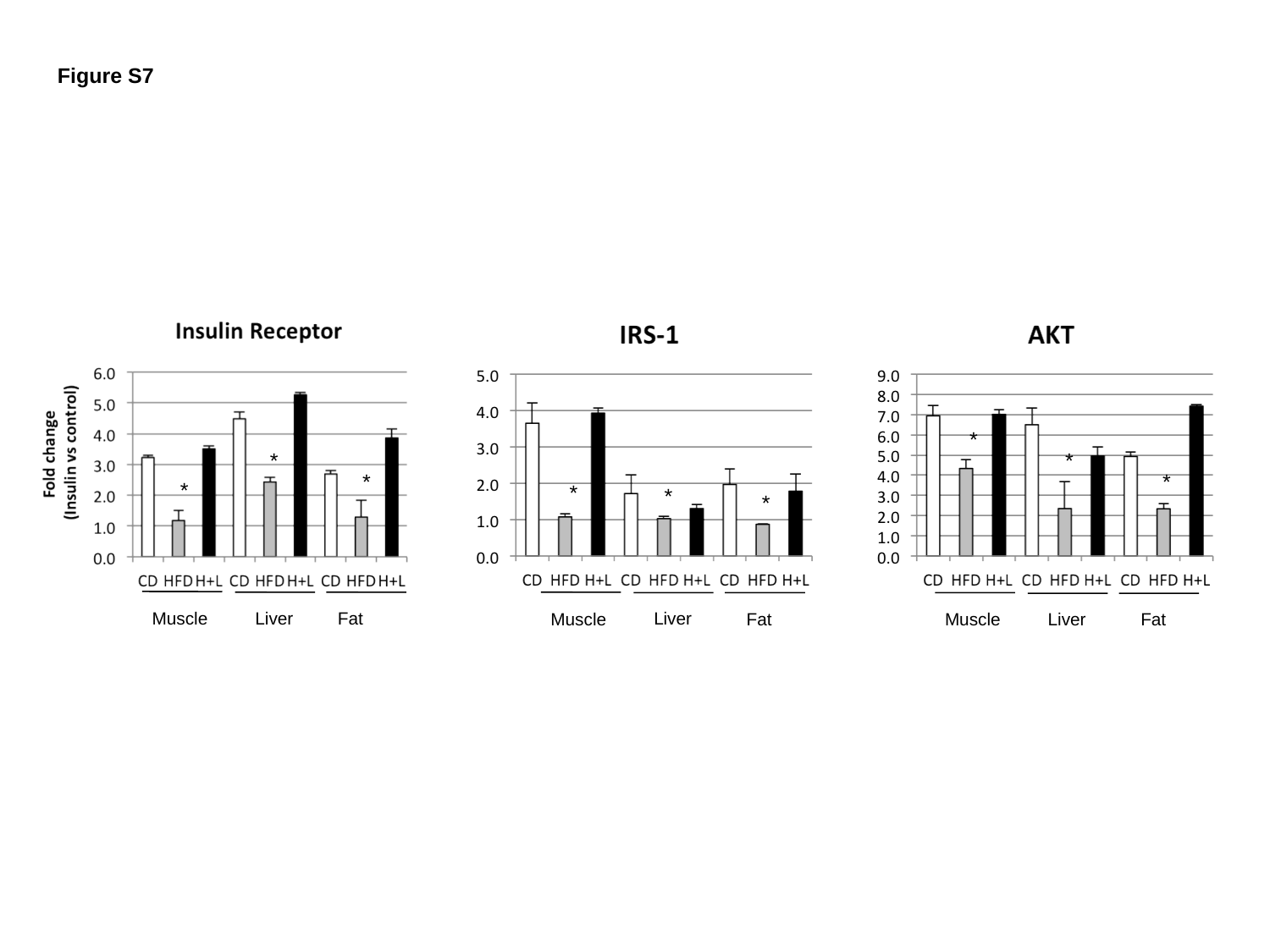

Figure S7
*
*
*
*
*
*
*
*
*
Liver
Muscle
Fat
Liver
Muscle
Fat
Liver
Muscle
Fat

Supplement: Figure S7 — Leucine supplementation normalizes phosphorylation of IR, IRS1Y and AKT in muscle, liver and PG fat. Phosphorylation was evaluated by western blot using specific antibodies for each protein. Quantification was done with Quantity one software (BioRad). Graphs represent the fold change in phosphorylation stimulated by insulin vs the non treated control and normalized by densities of the total proteins. N = 5 samples per group were quantified and western blots were repeated two times.*P<0.05 vs CD. (PPT) [file pone.0021187.s007.ppt]

## Slide 1
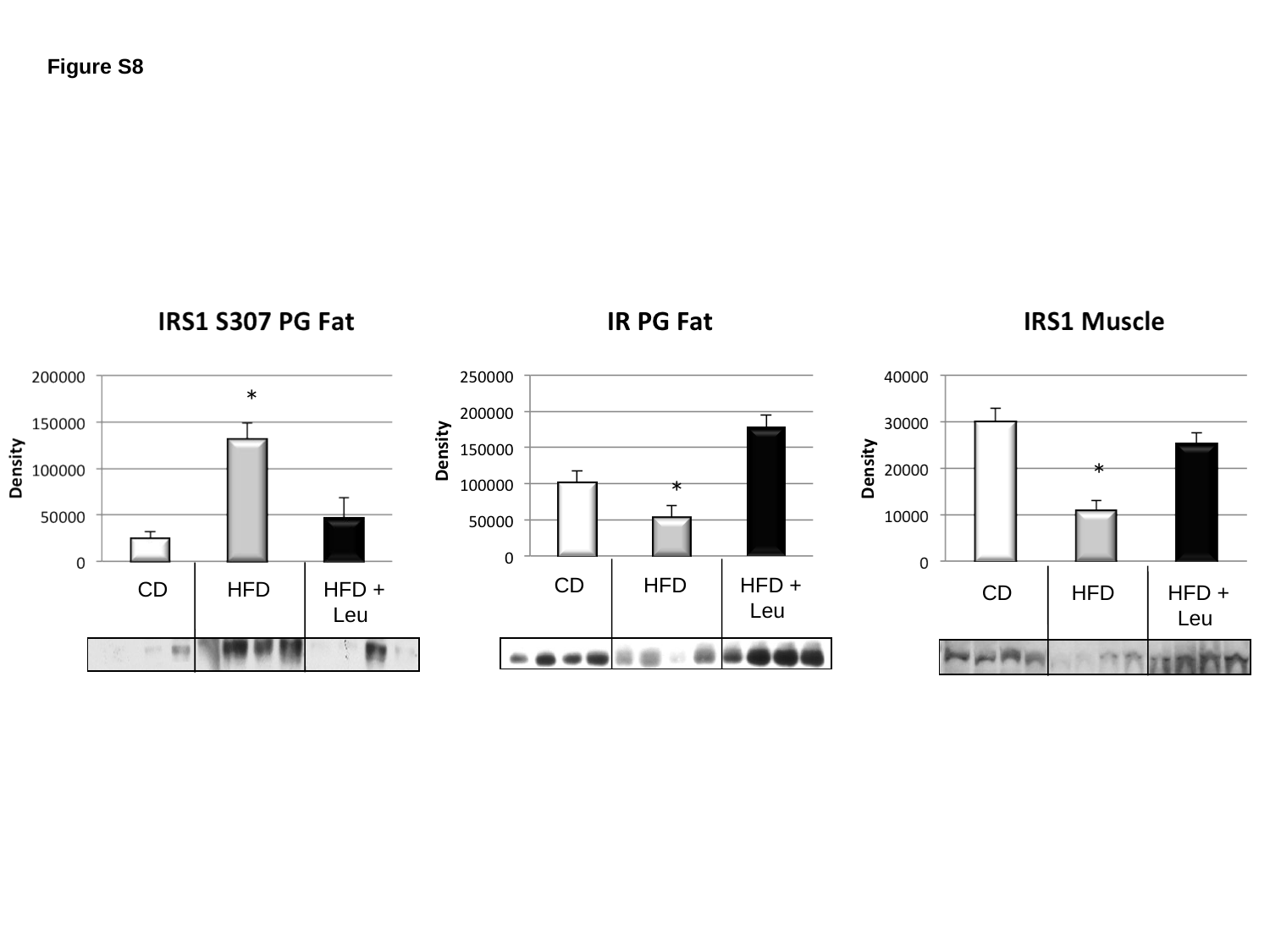

Figure S8
*
*
*
CD HFD HFD +
 Leu
CD HFD HFD +
 Leu
CD HFD HFD +
 Leu

Supplement: Figure S8 — Leucine supplementation normalizes protein expression of IR and reduces IRS1 S307 phosphorylation in fat and also normalizes IRS1 in muscle. Protein expression of IRS1 in muscle, IR in PG fat and IRS1 S307 phosphorylation was evaluated by western blot and quantified using Kodak software. n = 5 samples per group.*P<0.05. (PPT) [file pone.0021187.s008.ppt]

## Slide 1
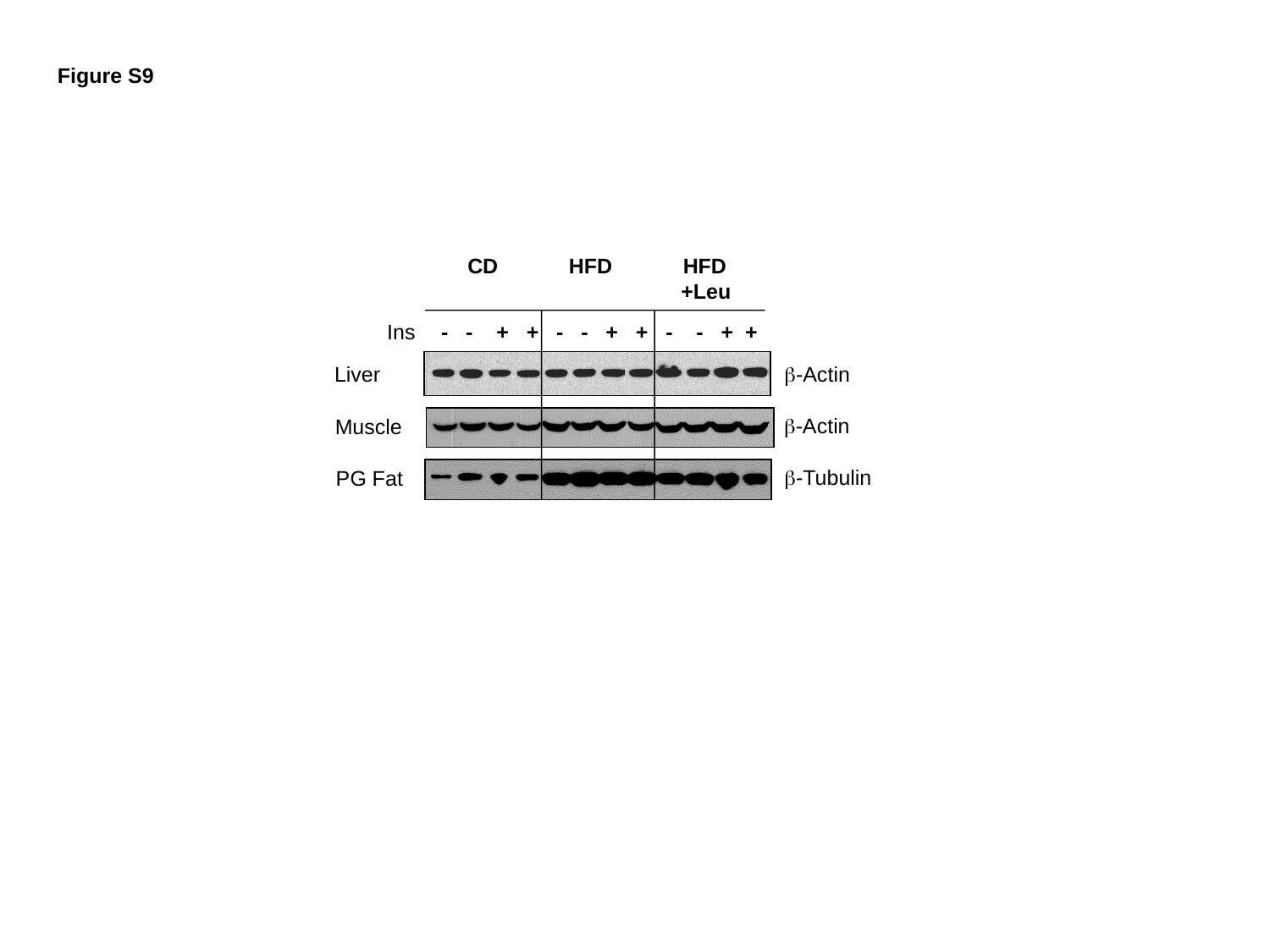

Figure S9
CD HFD HFD
 +Leu
Ins
- - + + - - + + - - + +
β-Actin
Liver
β-Actin
Muscle
β-Tubulin
PG Fat

Supplement: Figure S9 — Loading controls for Western blot on liver, muscle and PG fat. After 8 weeks on the different diets, mice were injected i.v. with insulin (5 U per mouse) or saline, and tissues were harvested 5 minutes later. Tissue protein lysates (20 mg) were run on SDS-PAGE and subjected to western blot using antibodies directed against b-actin for liver and muscle or b-tubulin for PG fat. 5 animals per group were used and the experiments were repeated 2 times and the blots were repeated 2 times. (PPT) [file pone.0021187.s009.ppt]

## Slide 1
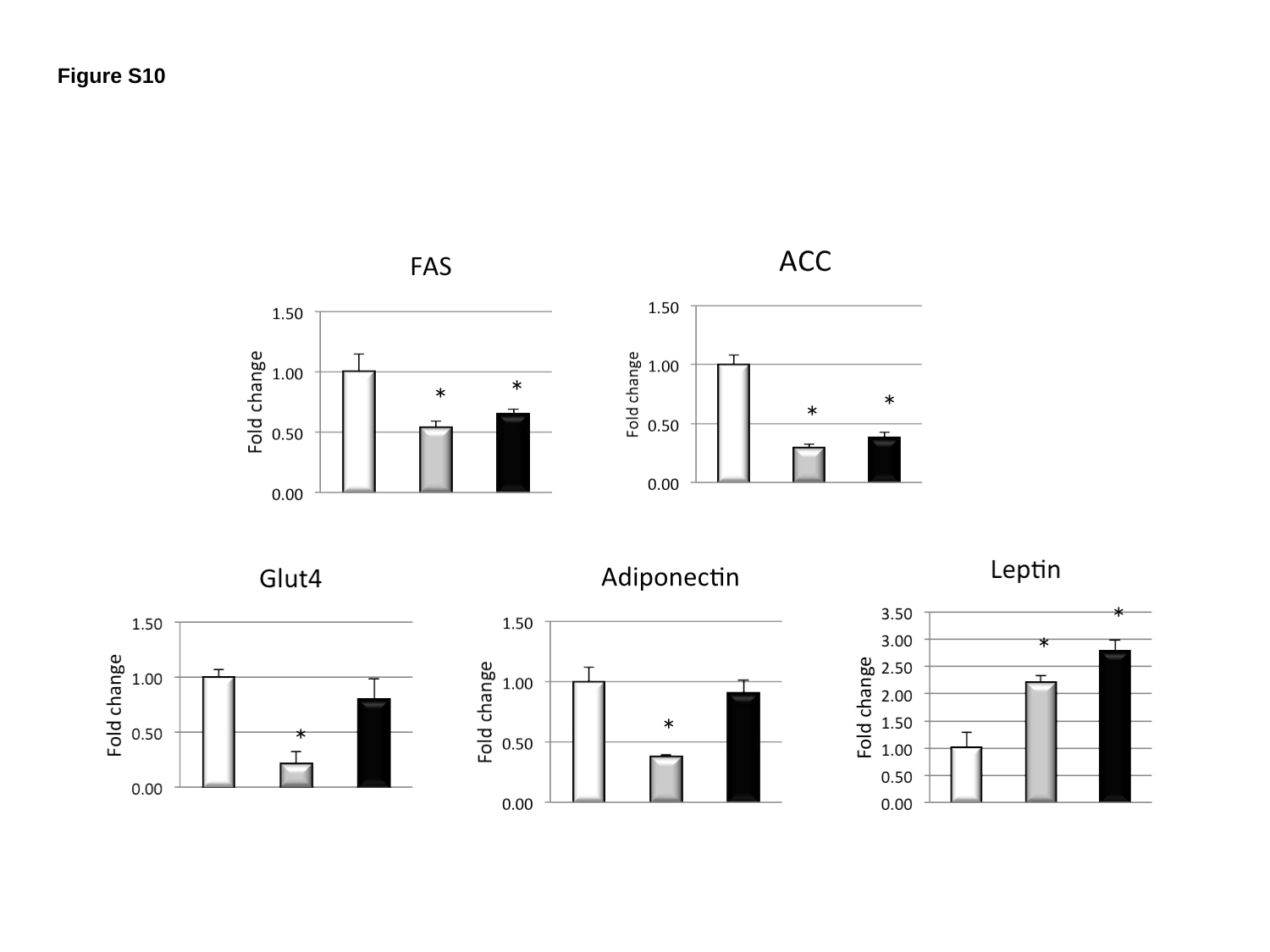

Figure S10
*
*
*
*
*
*
*
*

Supplement: Figure S10 — Leucine supplementation normalizes some alterations in metabolic visceral fat gene expression induced by HFD. mRNA was extracted from 200 mg perigonadal fat and subjected to quantitative real time PCR. Gene expression was normalized against TATA-binding protein (TBP). 5 fat depots per group were used. *P<0.05 vs CD. (PPT) [file pone.0021187.s010.ppt]
