# Supplementary material for: Dietary Leucine - An Environmental Modifier of Insulin Resistance Acting on Multiple Levels of Metabolism
Source: PLoS One. 2011 Jun 22;6(6):e21187. doi: 10.1371/journal.pone.0021187 (PMC3120846; doi:10.1371/journal.pone.0021187)
Supplement: Figure S3 — Leucine supplementation does not change leptin, adiponectin, triglyceride, glucagon or c-peptide serum levels. Serum levels were evaluated by ELISA in 5 samples per group in random fed animals at 8 weeks after the initiation of each dietary condition. Values are means ± SE. (PPT) [file pone.0021187.s003.ppt]

## Slide 1
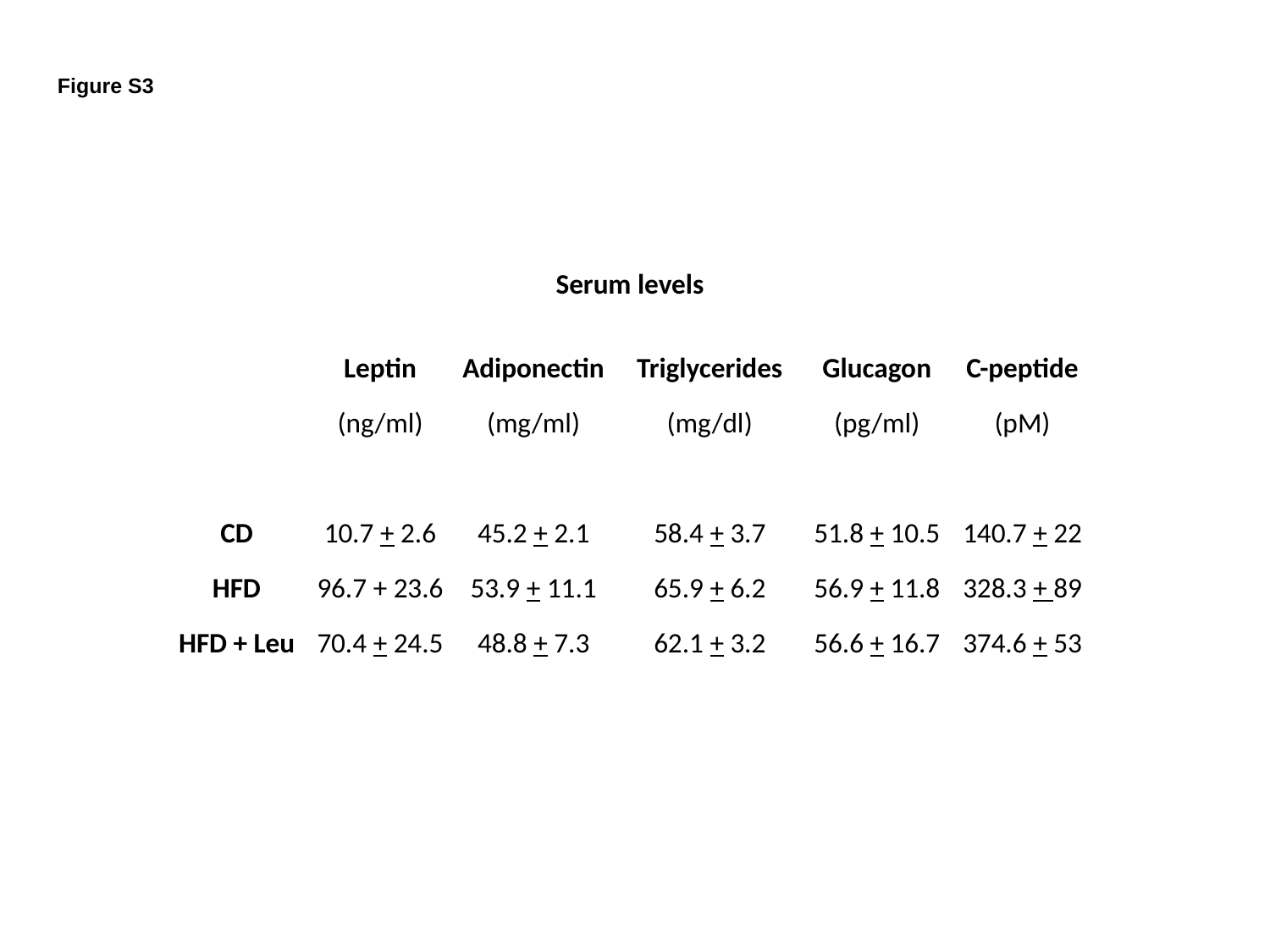

Figure S3
| Serum levels | | | | | |
| --- | --- | --- | --- | --- | --- |
| | Leptin | Adiponectin | Triglycerides | Glucagon | C-peptide |
| | (ng/ml) | (mg/ml) | (mg/dl) | (pg/ml) | (pM) |
| | | | | | |
| CD | 10.7 + 2.6 | 45.2 + 2.1 | 58.4 + 3.7 | 51.8 + 10.5 | 140.7 + 22 |
| HFD | 96.7 + 23.6 | 53.9 + 11.1 | 65.9 + 6.2 | 56.9 + 11.8 | 328.3 + 89 |
| HFD + Leu | 70.4 + 24.5 | 48.8 + 7.3 | 62.1 + 3.2 | 56.6 + 16.7 | 374.6 + 53 |
