# Supplementary material for: Dietary Leucine - An Environmental Modifier of Insulin Resistance Acting on Multiple Levels of Metabolism
Source: PLoS One. 2011 Jun 22;6(6):e21187. doi: 10.1371/journal.pone.0021187 (PMC3120846; doi:10.1371/journal.pone.0021187)
Supplement: Figure S4 — Leucine supplementation in chow diet does not change metabolic parameters. Body weight, GTT, liver histology and liver gene expression were analyzed in mice on a CD, CD+Leu, HFD and HFD+Leu. (PPT) [file pone.0021187.s004.ppt]

## Slide 1
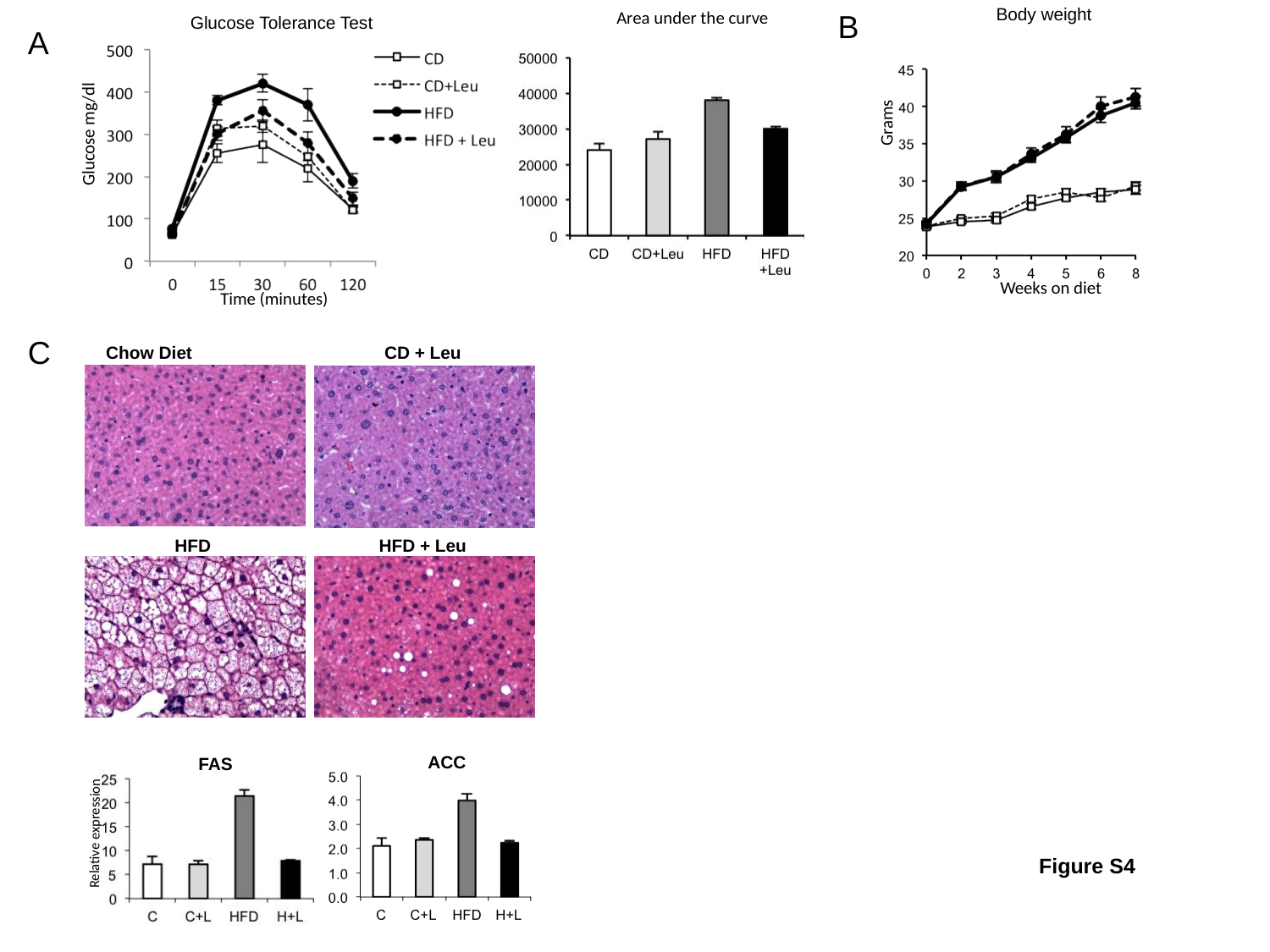

B
Body weight
Glucose Tolerance Test
Area under the curve
A
Grams
Glucose mg/dl
Weeks on diet
Time (minutes)
C
Chow Diet
CD + Leu
HFD
HFD + Leu
ACC
FAS
Relative expression
Figure S4
