# Supplementary material for: Dietary Leucine - An Environmental Modifier of Insulin Resistance Acting on Multiple Levels of Metabolism
Source: PLoS One. 2011 Jun 22;6(6):e21187. doi: 10.1371/journal.pone.0021187 (PMC3120846; doi:10.1371/journal.pone.0021187)
Supplement: Figure S11 — Other changes in metabolites induced by leucine supplementation. After 8 weeks on each diet, serum, hindlimb skeletal muscle, liver and perigonadal fat were obtained, extracted and subjected to non-targeted metabolomic analysis by UHPLC-MS/MS and GC-MS (Metabolon). Box-and-whisker boxplots of relative levels are shown for A) NADH and Tryptophan metabolism, B) Polyol Pathway, C) Alpha-hydroxybutyrate in liver and serum. 3-9 samples per group were used. *P<0.05. (PPT) [file pone.0021187.s011.ppt]

## Slide 1
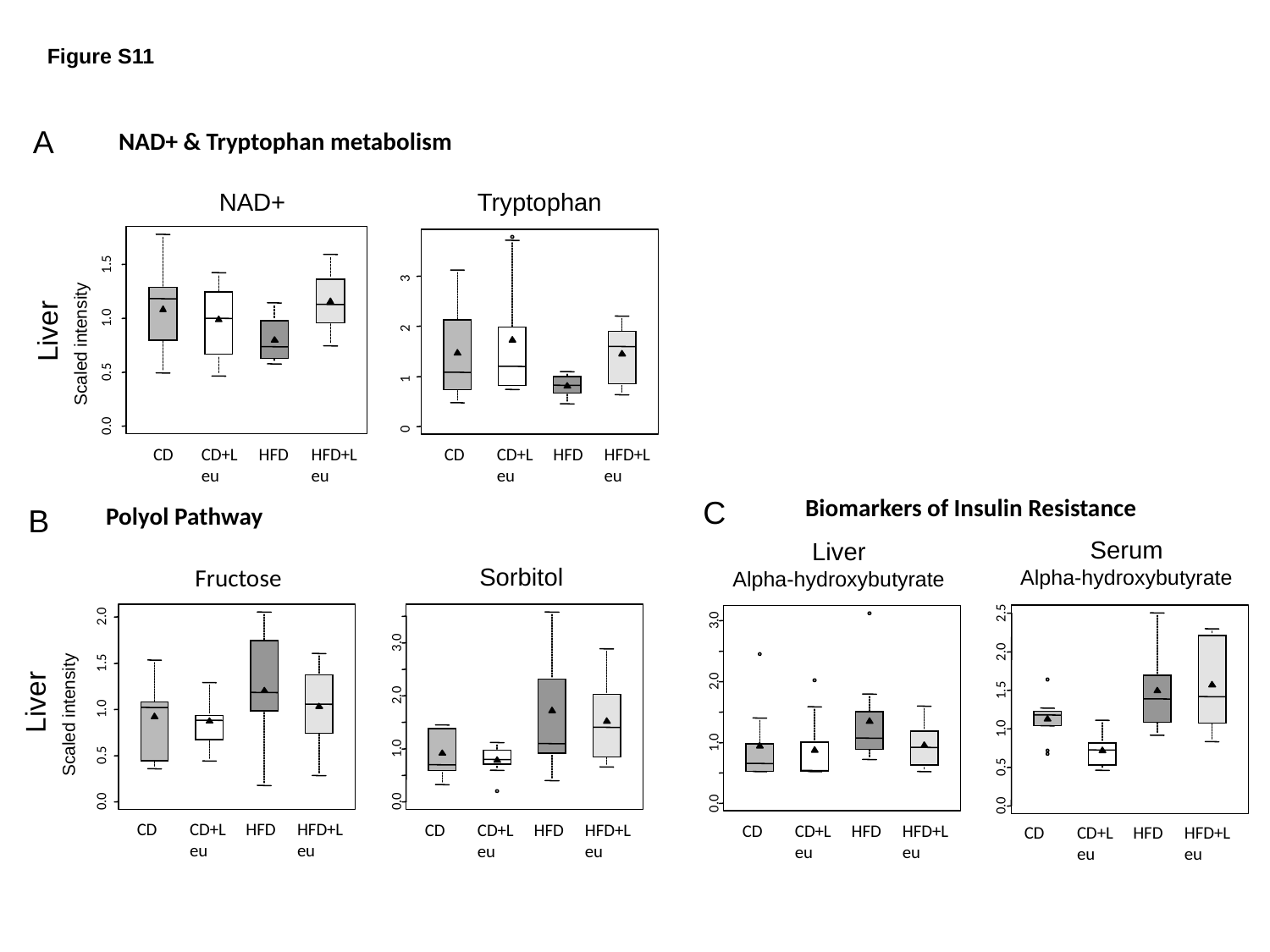

Figure S11
A
NAD+ & Tryptophan metabolism
NAD+
Tryptophan
1.5
3
Liver
1.0
2
Scaled intensity
0.5
1
0.0
0
CD
CD+Leu
HFD
HFD+Leu
CD
CD+Leu
HFD
HFD+Leu
Biomarkers of Insulin Resistance
C
Polyol Pathway
B
Liver
Alpha-hydroxybutyrate
Serum
Alpha-hydroxybutyrate
Sorbitol
Fructose
2.5
2.0
3.0
3.0
2.0
1.5
2.0
Liver
1.5
2.0
Scaled intensity
1.0
1.0
1.0
1.0
0.5
0.5
0.0
0.0
0.0
0.0
CD
CD+Leu
HFD
HFD+Leu
CD
CD+Leu
HFD
HFD+Leu
CD
CD+Leu
HFD
HFD+Leu
CD
CD+Leu
HFD
HFD+Leu
